# Phytoregionalisation of the Andean páramo

**DOI:** 10.7717/peerj.4786

**Published:** 2018-06-01

**Authors:** Gwendolyn Peyre, Henrik Balslev, Xavier Font

**Affiliations:** 1 Department of Civil and Environmental Engineering, Universidad de los Andes, Bogotá, Colombia; 2 Department of Bioscience, Aarhus University, Aarhus, Denmark; 3 Department of Plant Biology, Universitat de Barcelona, Barcelona, Spain

**Keywords:** Andes, Bioregionalisation, Clustering, Diagnostic species, Phytogeography, Phytosociology, Principal component analysis, Vascular plants

## Abstract

**Background:**

The páramo is a high-elevation biogeographical province in the northern Andes, known for its great biodiversity and ecosystem services. Because there have been very few biogeographic studies encompassing the entire province to date, this study aimed at conducting a phytogeographical regionalisation of the páramo. Specifically, (1) clustering analyses were conducted to identify the main phytogeographical units in the three altitudinal belts: sub-páramo, mid-páramo and super-páramo, and examine their diagnostic flora, (2) an ordination complemented the geo-climatic characterization of the obtained units and (3) a hierarchical classification transformation was obtained to evaluate the relationships between units.

**Methods:**

The study area included the entire Andean páramo range in northern Peru, Ecuador, Colombia and Venezuela. The analyses were based on 1,647 phytosociological plots from the *VegPáramo* database. The K-means non-hierarchical clustering technique was used to obtain clusters identifiable as phytogeographical units, and the Ochiai fidelity index was calculated to identify their diagnostic species. A principal component analysis was conducted to obtain the geo-climatic characterization of each unit. Finally, the relationships between clusters were traced using a hierarchical plot-based classification.

**Results:**

Fifteen clusters were obtained, 13 natural and two artificial, of which two represented the sub-páramo, nine the mid-páramo and four the super-páramo. Even though data representativeness was a potential limitation to segregate certain sub-páramo and super-páramo units, the overall bioregionalisation was robust and represented important latitudinal, altitudinal and climatic gradients.

**Discussion:**

This study is the first to bioregionalise the páramo province based on a substantial widely distributed biological dataset, and therefore provides important novel scientific insight on its biogeography. The obtained phytogeographical units can be used to support further research on the páramo at smaller scale and on the humid Neotropical high-elevation ecosystems at broader-scale. Finally, several units were highlighted in our results as particularly worthy of further scientific and conservation focus.

## Introduction

The Andean páramo is defined as a biogeographical province (Morrone, 2014) of high elevation ecosystems located above the montane treeline in the mountains of northern Peru, Ecuador, Colombia and Venezuela (Luteyn, 1999). With a geographic distribution over almost 20° latitude around the equator and 2,000 m elevation, the páramo constitutes an excellent model for tropical alpine regions worldwide (Nagy & Grabherr, 2009; Sklenář, Hedberg & Cleef, 2014). The recent orogeny of the northern Andes with a last uplift during the Miocene created an archipelago of continental biogeographic islands on mountain tops, which nowadays sustains the páramo ecosystems. The following glaciation dynamics of the Quaternary periodically formed either biogeographical barriers or vast available niches in the area, hence accelerating taxonomic diversification especially for still organisms such as plants (Hughes & Eastwood, 2006; Anthelme et al., 2014). As a result, the páramo is today considered the fastest and coolest evolving biodiversity hotspot (Madriñán, Cortés & Richardson, 2013), and also the floristically richest tropical alpine province, counting about 5,000 plant species spread over more than 500 plant communities (Rangel-Churio, 2000, 2015; Sklenář, Hedberg & Cleef, 2014). Apart from its remarkable biodiversity and high endemism, estimated at 60% of its flora (Luteyn, 1999), the páramo is further known for contributing essential ecosystem services to local communities and cities such as Quito and Bogota, among which water provision and climate regulation, through carbon stocking, are particularly important (Buytaert, Cuesta-Camacho & Tobón, 2011; Farley et al., 2013). Anthropogenic activities, including agriculture, farming and mining, have rapidly increased in extent and intensity for the past 50 years and now challenge the ecological resistance and resilience of the páramo (Vásquez, Balslev & Sklenář, 2015). Climate change is also becoming a critical threat, especially near the nival altitudinal belt where species migration is limited (Morueta-Holme et al., 2016), although there is yet much to understand about the adaptation capacity of páramo species. To date, there is extensive research on páramo ecology that translates into numerous works on flora, fauna, biotope and ecosystems (Ramsay & Oxley, 1996; Molinillo & Monasterio, 2002; Vásquez, Balslev & Sklenář, 2015). However, other important and related research fields, such as biogeography, remain understudied in the páramo and in tropical alpine regions in general (Hoorn et al., 2010; Anthelme & Lavergne, 2018). This is in part due to our incomplete knowledge of tropical taxa, the lack of geographically extensive biological datasets and the difficulties in accounting for environmental heterogeneity in topographically complex areas (Stein, Gerstner & Kreft, 2014; Engemann et al., 2015). Nonetheless, thanks to the recent improvements in tropical biological databases and atmospheric science (Peyre et al., 2015; Karger et al., 2017), promising new research is expected on páramo and tropical alpine biogeography.

Biogeography is the field that studies spatial patterns of biodiversity at a wide range of spatial and temporal scales (Olson et al., 2001; Morrone, 2014). In this context, the bioregionalisation approach, which aims at understanding how natural areas that are characterised by homogeneous compositions of species and delimited by biogeographical boundaries occur and coexist, has proven very resourceful and receives important scientific attention today (Antonelli, 2017; Ficetola, Mazel & Thuiller, 2017). Although related, this approach goes beyond the simple measure of beta diversity that quantifies species turnover (species replacement) or nestedness of species assemblages (species loss) in an environment or spatially defined pattern of environments (Tuomisto, 2010). In fact, bioregionalisation solely uses species occurrences and sometimes abundances to define species assemblages and identify biogeographic units, without considering spatial continuity and distance (Kreft & Jetz, 2010; Legendre & de Cáceres, 2013; Vilhena & Antonelli, 2015; Antonelli, 2017). Therefore, each biogeographic unit is defined by a list of coexisting species and characterised by its dominant and diagnostic species, which act as ecological or biogeographical indicators of this particular unit (De Cáceres & Wiser, 2012). Methods commonly used to conduct bioregionalisation studies usually rely on evolutionary, distribution or macroecological data, and call for an array of techniques such as similarity, clustering or more recently networks (Vilhena & Antonelli, 2015). Such studies have received much attention for their appeal in serving many scientific purposes, including evolution research, for example evaluating niche-conservatism within large areas (Crisp et al., 2009) and predicting biodiversity variation under climate change (Salazar, Nobre & Oyama, 2007). Moreover, bioregionalisation serves conservation science, for instance by allowing to target species-rich and threatened areas similarly to the biodiversity hotspot approach (Mittermeier et al., 2011; Bloomfield, Knerr & Encinas-Viso, 2018).

Although few broad-scale bioregionalisations have already been conducted in the wide Andes based on species distribution data, either faunistic (Morrone, 2015) or floristic (Cuesta et al., 2017), and vegetation-based indices (Josse et al., 2009), the data used in such studies might not be considered sufficiently representative to characterize the páramo province in detail. Moreover, there have been several attempts to recognise biogeographical units within the páramo province, using small-scale studies (up to national scale) based on biological data, among which species turnover quantifications, parsimony analyses of endemicity and vegetation classifications (Ramsay, 1992; Rangel-Churio, 2000; Sklenář, 2000; Beltrán et al., 2009; Cuello & Cleef, 2009; Londono, Cleef & Madriñán, 2014). However, no bioregionalisation study encompassing the entire páramo province and relying on a representative and substantial biological dataset has been conducted to date. In this context, the recent *VegPáramo—*the flora and vegetation database for the Andean páramo (Peyre et al., 2015)—and the 3,000 vegetation plots it contains, represents important distribution data for coexisting páramo plant species, widely distributed throughout the biogeographical province. Such new data availability announces potential advances for the floristic bioregionalising of the páramo into phytogeographical units. Finally, the consequent results could have further repercussions on both páramo research, e.g., providing new delimitations to understand biogeographical boundaries of historical, geographic, biotic and abiotic nature, but also conservation, e.g., identifying geographically restricted phytogeographical units with specialised and endemic flora that should benefit from priority management (Olson et al., 2001; Kreft & Jetz, 2010).

Our main objective in this study was to conduct a broad-scale bioregionalisation of the páramo province based on its flora. Specifically, we (1) relied on the commonly used clustering approach to classify and identify main páramo phytogeographical units (González-Orozco et al., 2014; Ebach et al., 2015) and then characterise them by their diagnostic species, (2) used an ordination to associate the obtained units to spatial and bioclimatic data and obtain their geo-climatic characterisation and (3) transformed the clustering results into a hierarchical classification to establish and quantify the similarity between phytogeographical units.

## Study Area

**Figure 1:**
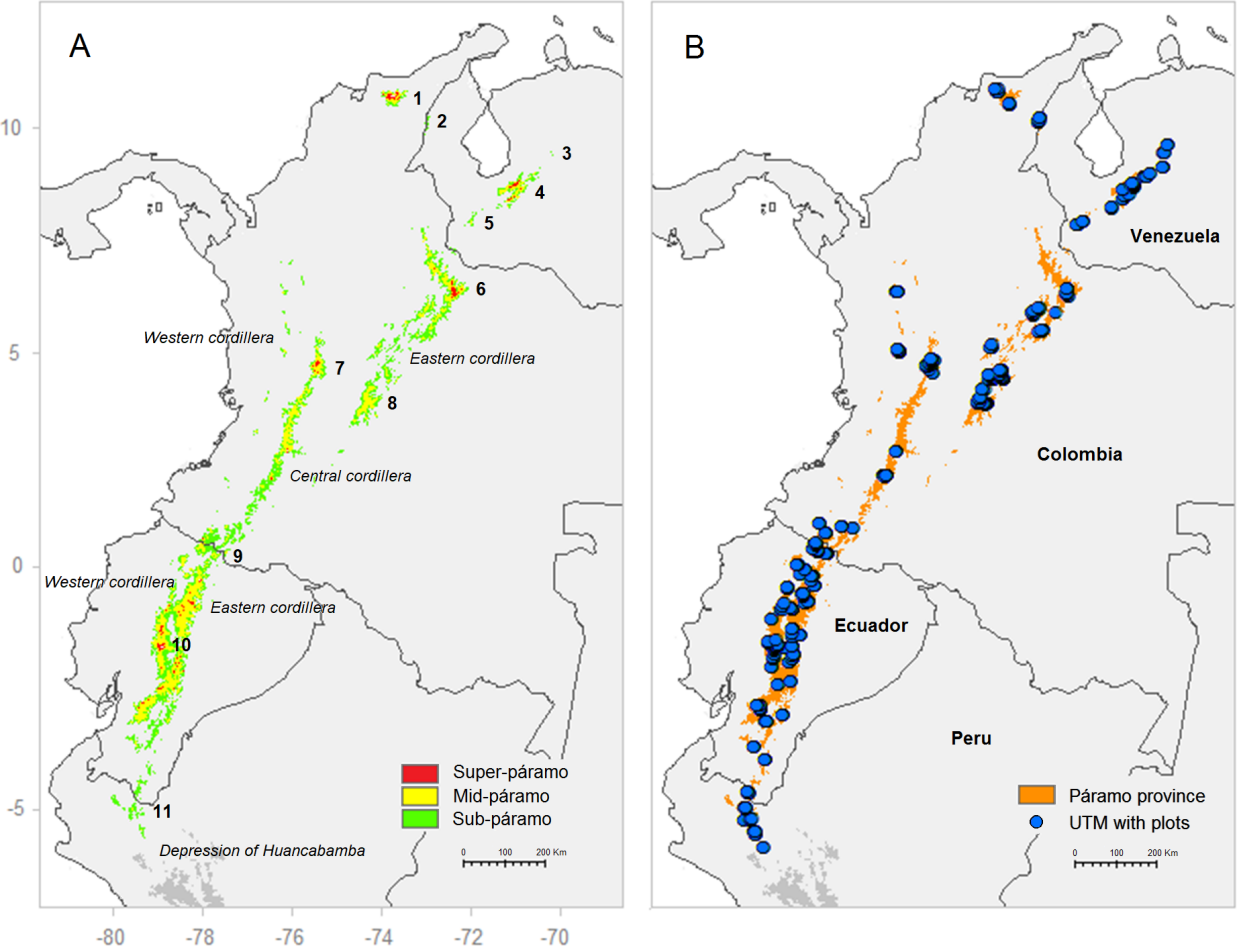
(A) Potential distribution of the páramo province in the northern Andes based on their approximate elevation ranges; (B) distribution of the vegetation plots (1 km UTM) used in this study. Sub-páramo 3,000–3,500 m; Mid-páramo 3,500–4,200 m; Super-páramo 4,200–5,000 m. *Main páramo areas (numbers from north to south): 1. Sierra Nevada de Santa Marta; 2. Sierra de Périja; 3. Guaramacal; 4. Cordillera de Merida; 5. Táchira; 6. Cocuy; 7. Nevados; 8. Sumapaz; 9. Carchi; 10. Chimborazo; 11. Amotape–Huancabamba zone*.

The study area covered the entire Andean páramo in South America stretching from northern Peru, over Ecuador and Colombia to Venezuela (Fig. 1A). For simplicity, the depression of Huancabamba (∼6°S–79°W) was used as southern limit of the páramo province because it is known as a biogeographical barrier for many Andean plant taxa and a differential area between the humid páramo and dry puna climatic provinces (Luteyn, 1999; Weigend, 2002). Nonetheless, the authors were aware that other studies limited the páramo further south and included microclimatic areas influenced by Amazonian humidity, for example the Bolivian Yungas (García & Beck, 2006). In this study, the páramo was considered to be confined to the Amotape–Huancambamba zone in northern Peru, an isolated mountain area shared with southern Ecuador. Further north, in central and northern Ecuador, the páramo is found on both the Eastern and Western Cordilleras that further divide in Colombia into the Eastern, Central and Western Cordilleras. Moreover, the Sierra of Périja and Sierra Nevada de Santa Marta isolated mountain ranges in northeastern Colombia (∼11°N–74°W) sustains the northernmost South American páramo. Finally, in Venezuela, the páramo occurs mostly in the cordillera of Merida as well as other smaller mountain areas such as the Sierra de Périja, shared with Colombia. The study area was limited to the Andean and Andean-relict páramos, excluding extra-Andean occurrences on Amazonian mountains, the coastal cordillera in Venezuela, and central American páramo areas. Finally, the entire páramo altitudinal range (∼3,000–5,000 m) was considered, including the three traditionally described altitudinal belts: (1) the lower ecotone with montane forests, or *sub-páramo* (∼3,000–3,500 m), (2) the intermediate páramo proper, referred here as *mid-páramo* (∼3,500–4,200 m) and (3) the higher *super-páramo* (∼4,200–5,000 m) (Cleef, 1981; Luteyn, 1999).

## Methods

### Vegetation data

This study was based on a dataset of 2,853 vegetation plots fitting the study area, sampled with the phytosociological method (Braun-Blanquet, 1951) and obtained from the *VegPáramo* database (www.vegparamo.com). All taxa were previously checked for synonymy with the *VegPáramo* taxon list, which contains over 15,000 plant names from the northern Andes that are frequently updated using *the Plant List* (www.theplantlist.org) and *Tropicos* (www.tropicos.org). The initial dataset included plots proceeding from 38 different sources and sampled by various authors over a period from 1981 to 2014. Because these plots were sampled in many different vegetation types, as often stated by their original author, the dataset was rather heterogeneous in terms of vegetation represented, including meadows, bogs, grasslands, giant rosette vegetation, shrublands, bamboo vegetation and forests among others. Moreover, because all plots were sampled with the phytosociological method, which instructs that plot size should vary according to vegetation physiognomy and composition (Ozenda, 1982), the dataset presented a wide range of plot sizes, from 1 to 100 m^2^, which we believed with our data handling (see below) should not affect our analyses. Regarding floristic content, each plot included a list of vascular, and sometimes non-vascular, plant species occurrences with their cover coefficients: + <1%, 1 >5%, 2 <25%, 3 <50%, 4 <75% and 5 >75%. Finally, plots were georeferenced using the UTM coordinate system at a 1 or 10 km^2^ resolution depending on the data source and the precision used in its plots’ geolocation.

In order to homogenize the species list, non-vascular plant species, unidentified taxa and supra-specific taxon names, i.e., genus, family, were removed. In addition, because some authors used sub-specific taxa, i.e., subspecies, variety, in their sampling and others did not, we summed all sub-specific taxa to the species level. Finally, species with low occurrence in the dataset (<2) were removed to reduce statistical noise. Because we aimed at characterising the main páramo biogeographical units under a macroclimatic and broad-scale geographical focus, we removed vegetation plots representing microclimatic or edaphic azonal vegetation, i.e., bogs, marshes, rocky vegetation or *Polylepis* relict forests, according to the original author’s description. Finally, to take into consideration the geographic sampling bias and varying geolocation precision, we (1) removed plots with >1 km resolution and (2) conducted a preferential geographic stratified resampling at 15 plots per 1 km UTM unit and uniformly distributed with elevation, divided into 200 m altitudinal strata (Knollová et al., 2005). These consecutive reductions lead to a final dataset of 1,647 plots for 1,724 species (Fig. 1B). Finally, to minimise the effect of subjective estimates in species cover, characteristic of phytosociological sampling, the phytosociological scale was transformed into a presence/absence binary scale (Kočí, Chytrý & Tichý, 2003).

### Non-hierarchical clustering and floristic characterisation

The following statistical analyses were carried out using the Ginkgo program of the b-VegAna application set (biodiver.bio.ub.es/veganaweb; Bouxin, 2005). The two main clustering methods available, hierarchical and non-hierarchical, apply partitioning to a dataset with an increasing number of clusters, however, the former generates a tree while the latter does not. Although a hierarchical classification on vegetation plot data might seem easier to interpret than a non-hierarchical one, one disadvantage is that plots that are assigned to a cluster at division state *n* cannot switch to a non-directly descendent cluster at the division *n* + 1, making the assignment permanent and the clustering rigid (De Cáceres & Wiser, 2012). On the contrary, non-hierarchical clustering is more flexible and might not even yield the same plot assignment result for each iteration of the same partition (Tichý, Chytrý & Smarda, 2011), which makes it more difficult to interpret but more precise and fitted to the data. Because our dataset included certain diversity of vegetation types, whose proportions were probably neither balanced nor representative of real landscapes due to sampling bias, we opted for a non-hierarchical clustering analysis to generate the páramo bioregionalisation, so to ensure independence between clusters of different partitions.

First, the dataset was converted into a plot-distance matrix using the Bray–Curtis distance, a widely used coefficient for community data in ecology (Bray & Curtis, 1957; Tichý et al., 2010). Second, the matrix was classified using an unsupervised non-hierarchical agglomerative K-means clustering technique, a particularly appropriate approach when working with heterogeneous datasets (MacQueen, 1967; Chytrý et al., 2002; De Cáceres & Wiser, 2012). This clustering technique is based on the random setting of clusters’ seeds and requires for the desired number of clusters to be previously established; in this case, set for partitions of 2–100 clusters with a one-unit pace increase (an exhaustive number given to necessarily include the optimal partition). For each partition, 10,000 iterations were conducted in order to obtain the best clusters configuration available for the páramo matrix. The resulting clusters, could be assimilated to a group of vegetation plots that shared affinities in terms of plant assemblages, dominant species and diagnostic ones (Chytrý et al., 2002). Then, the widely used, context-independent Ochiai indicator index (OI) was calculated to obtain the diagnostic value of each species within each cluster of each partition (Ochiai, 1957; De Cáceres, Font & Oliva, 2008). OI values were thresholded at 0.3 for species to be considered diagnostic of a specific cluster. Furthermore, cluster preference was checked, ensuring that the OI value considered diagnostic at least doubled the next highest OI value for the same species in a different cluster. Finally, particular attention was paid to rare species with low OI value but high exclusivity (Peyre & Font, 2011).

The optimal partition with the best cluster division was selected to represent the main páramo phytogeographical units. To do so, it is usual to rely on statistical criteria, such as the silhouette or pseudo-F, whose values depend on the number and scores of diagnostic species (De Cáceres & Wiser, 2012), even though there is no generally approved criterion to date (Tichý et al., 2010). However, the heterogeneity in our dataset implied that, with increasing partitions, certain units differentiated and separated faster than others, which prevented from using a strictly statistically based criterion that would compare similar speed divisions. Therefore, we built a subjective criterion that ensured sufficient variability, i.e. number of plots—set at 20, and floristic characterisation, i.e. number of diagnostic species—set at five, for every cluster. The optimal partition was hence defined as the most advanced clustering partition that met such criterion. Given the subjective character of the selection criterion employed, we provided five plots for each cluster to be used as seeds in case researchers are interested in reproducing our analyses with a supervised K-means clustering (Supplemental information 1). The optimal partition obtained therefore presented a certain number of *natural* clusters corresponding to an aggregation of vegetation plots with similar species content. However, some clusters might have not met these characteristics but instead corresponded to an aggregation of plots lacking the floristic similarity required to fit into a natural cluster but representing various vegetation types in too small numbers to create a new cluster. Such clusters should be qualified as *artificial* and are a common side effect of statistical classifications conducted on heterogeneous vegetation data, because each plot must fit into a cluster (Andrés & Font, 2011).

### Bioclimatic data and geo-climatic characterisation

To correlate the natural cluster previously obtained with geo-climatic data, we relied on several climatic variables: annual mean temperature, temperature annual seasonality, annual mean precipitation, precipitation annual seasonality and cloud annual cover. The temperature and precipitation variables were obtained at a resolution of 30 arc-seconds (∼1 km) from the CHELSA project 1.2, *Climatologies at High resolution for the Earth’s Land Surface Areas* (http://chelsa-climate.org), which enhances bioclimatic data quality in tropical areas (Karger et al., 2017). The cloud variable was also obtained at a resolution of 30-arc-seconds from the EarthEnv Project 1 (http://www.earthenv.org; Wilson & Jetz, 2016). Using the R 3.4.3 software, all variables were cropped at the vegetation data extent and elevation (Supplemental Information 2). Each variable was then extracted for the latitude and longitude decimal coordinates corresponding to each UTM central point of the plot data. Finally, a data-frame containing for each plot its UTM central decimal coordinates, elevation and a value for each of the five bioclimatic variables considered was created.

Then, a geo-climatic principal component analysis (PCA) of the plot data was conducted, analyzed and visualised after applying the optimal clustering partition. Therefore, every cluster could be assimilated to a páramo phytogeographical unit, characterised by its list of dominant and diagnostic species as well as its climate and geography.

### Hierarchical classification—similarity between phytogeographical units

One advantage of hierarchical clustering compared to non-hierarchical clustering is that it allows to understand the relationships within and between partitions thanks to the order of successive division (Tichý, Chytrý & Smarda, 2011). Since one of this study’s goals was to statistically quantify the relationships between the final clusters, the previously obtained K-means clustering results from partitions 2 to the optimal partition were transformed into a distance hierarchical classification. To do so, the Bray–Curtis distance was calculated, based on plot composition, for each pair of clusters of two successive K-means partitions, and then transformed into a similarity measure (*S* = 1 − *D*). All obtained values, corresponding to plot shifts and cluster forming with progressing divisions of the dataset, i.e., increasing partitions, were merged into a similarity matrix that quantified the relationships between the clusters of the optimal partition and all clusters from previous partitions. Then, a hierarchical divisive classification was built, using all similarity values superior to 0.1 (max. 1). Finally, this similarity tree up to the optimal partition was interpreted to (1) understand how easily the final clusters formed and segregated from the rest of the dataset, i.e., when no important plot exchange occurred and *S* values remained constantly high, and (2) evaluate their closeness to the other phytogeographical units.

## Results

**Figure 2:**
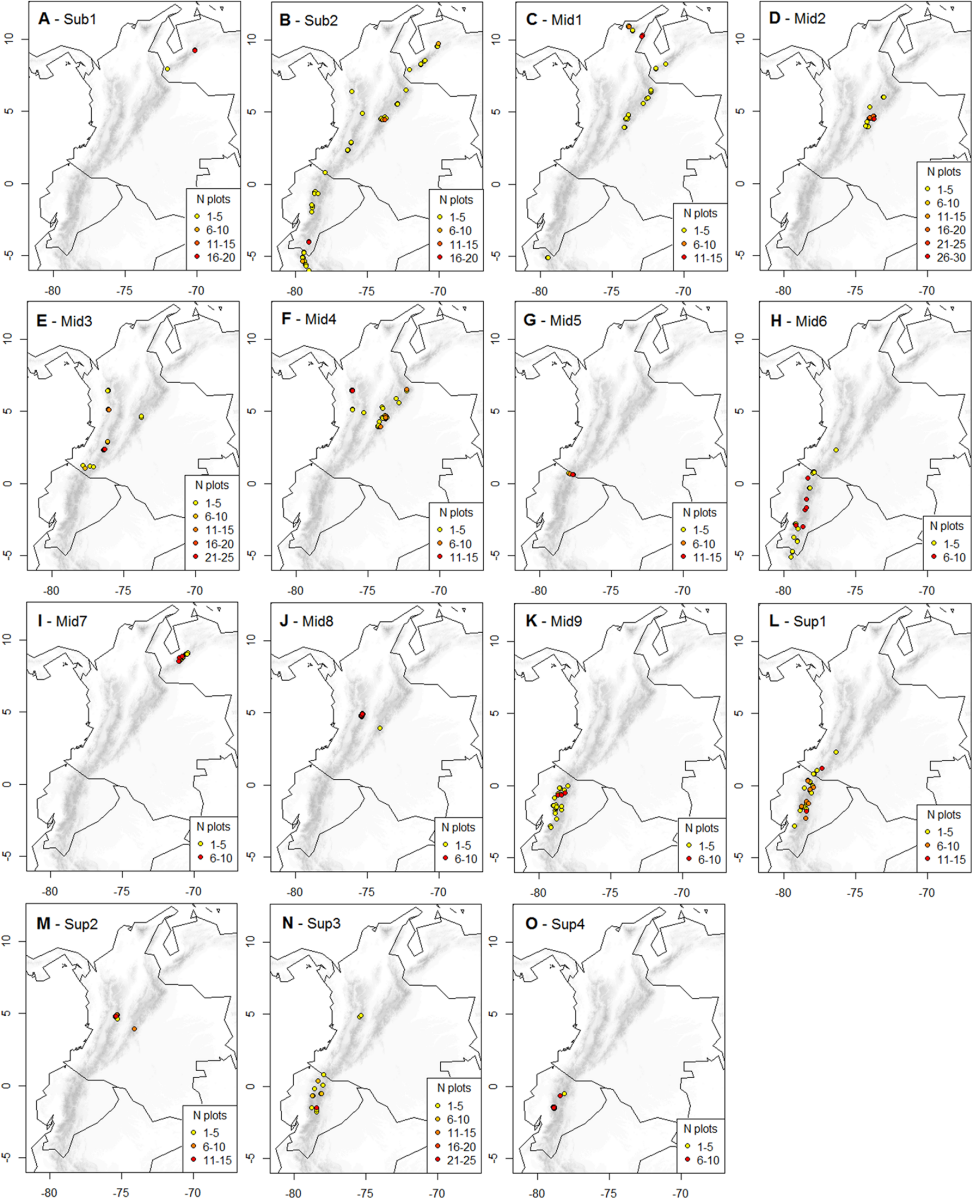
Geographic distribution of the K-means optimal partition in 15 clusters throughout the páramo province. For each cluster, vegetation plots are shown with their UTM central point’s decimal coordinates and their corresponding local abundance. Isolated plots, represented by UTM with one plot only and whose distribution is not directly adjacent to another occurrence UTM, were considered outliers and not represented. A complete list of the outlier plots is provided in Supplemental Information 3. (A) *Sub-1: Guaramacal sub-páramo, 0% outliers;* (B) *Sub-2: Widespread subpáramo, 8.9% outliers;* (C) *Mid-1: Périja-Santa Marta mid-páramo, 5.9% outliers;* (D) *Mid-2: Eastern cordillera mid-páramo, 2.9% outliers;* (E) *Mid-3: Central and western cordilleras mid-páramo, 3% outliers;* (F) *Mid-4: Mixed group of humid mid-páramo, 6.2% outliers;* (G) *Mid-5: Carchi mid-páramo, 1.8% outliers;* (H) *Mid-6: Ecuadorian mid-páramo, 2.9% outliers;* (I) *Mid-7: Venezuelan mid-páramo and lower super-páramo, 0% outliers;* (J) *Mid-8: The Nevados upper mid-páramo, 0%;* (K) *Mid-9: The Ecuadorian upper mid-páramo, 2.1% outliers;* (L) *Sup-1: Lower humid super-páramo, 0.6% outliers;* (M) *Sup-2: The Nevados super-páramo, 4.9% outliers;* (N) *Sup-3: Upper humid super-páramo, 1.9% outliers;* (O) *Sup-4: Upper dry Ecuadorian super-páramo, 1.6% outliers*.

**Figure 3:**
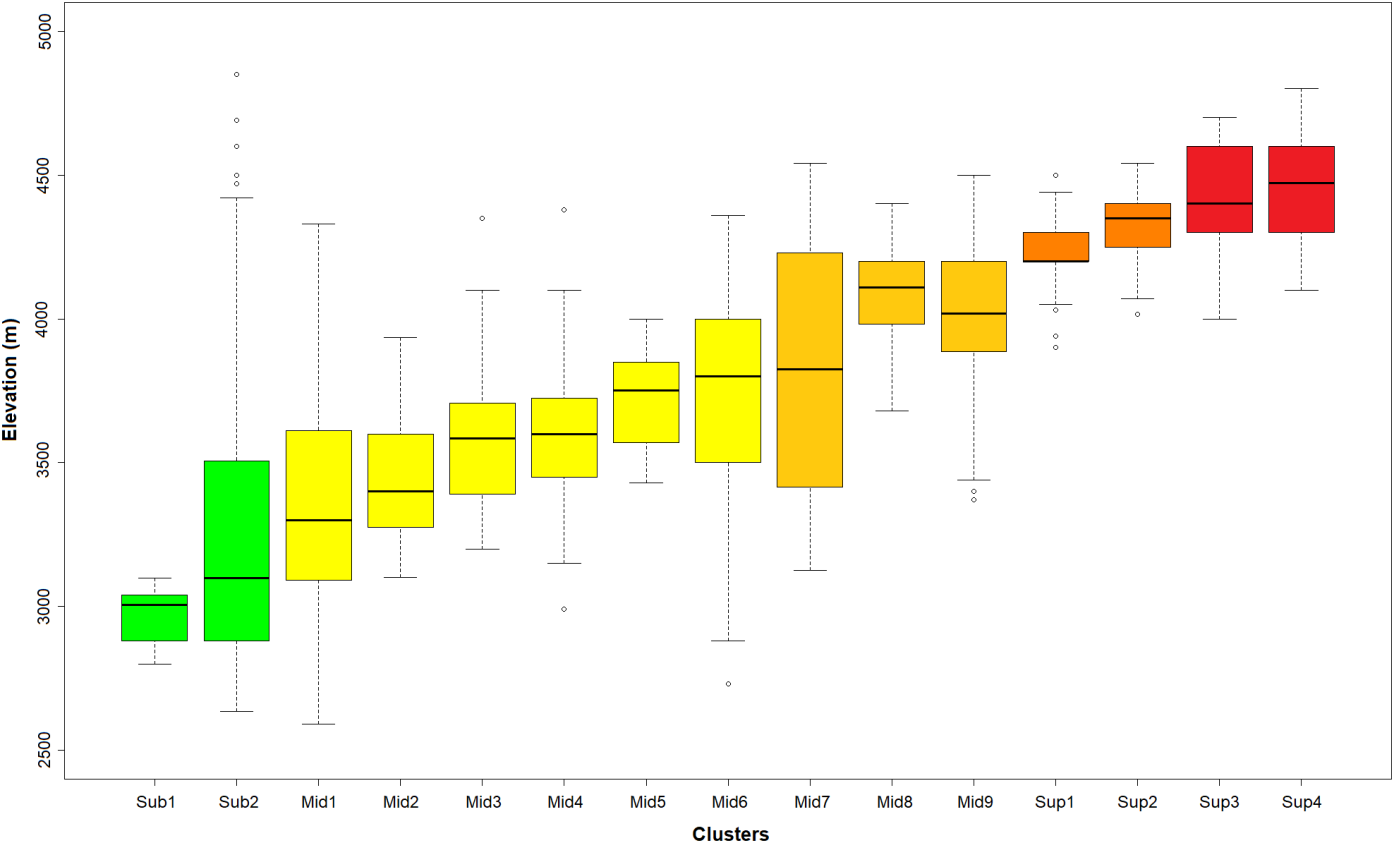
Distribution of the K-means optimal partition in 15 clusters along the altitudinal gradient. Outlier plots considered in Fig. 2 were also removed in this representation. In green: sub-páramo; in light yellow: mid-páramo; in dark yellow: upper mid-páramo; in orange: lower super-páramo; in red: upper super-páramo. *Sub-1: Guaramacal sub-páramo, Sub-2: Widespread sub-páramo, Mid-1: Périja-Santa Marta mid-páramo, Mid-2: Eastern cordillera midpáramo, Mid-3: Central and western cordilleras mid-páramo, Mid-4: Mixed group of humid mid-páramo, Mid-5: Carchi mid-páramo, Mid-6: Ecuadorian mid-páramo, Mid-7: Venezuelan mid-páramo and lower superpáramo, Mid-8: The Nevados upper mid-páramo, Mid-9: The Ecuadorian upper mid-páramo, Sup-1: Lower humid super-páramo, Sup-2: The Nevados super-páramo, Sup-3: Upper humid super-páramo, Sup-4: Upper dry Ecuadorian super-páramo*.

Based on the established selection criterion of most advanced partition with minimum 20 plots and five diagnostic species per cluster, we found the 15 clusters partition to be optimal. This partition included 13 natural clusters and two artificial clusters, distributed throughout the páramo province and showing elevation and climatic differentiation (Figs. 2 and 3).

### PCA results

**Figure 4:**
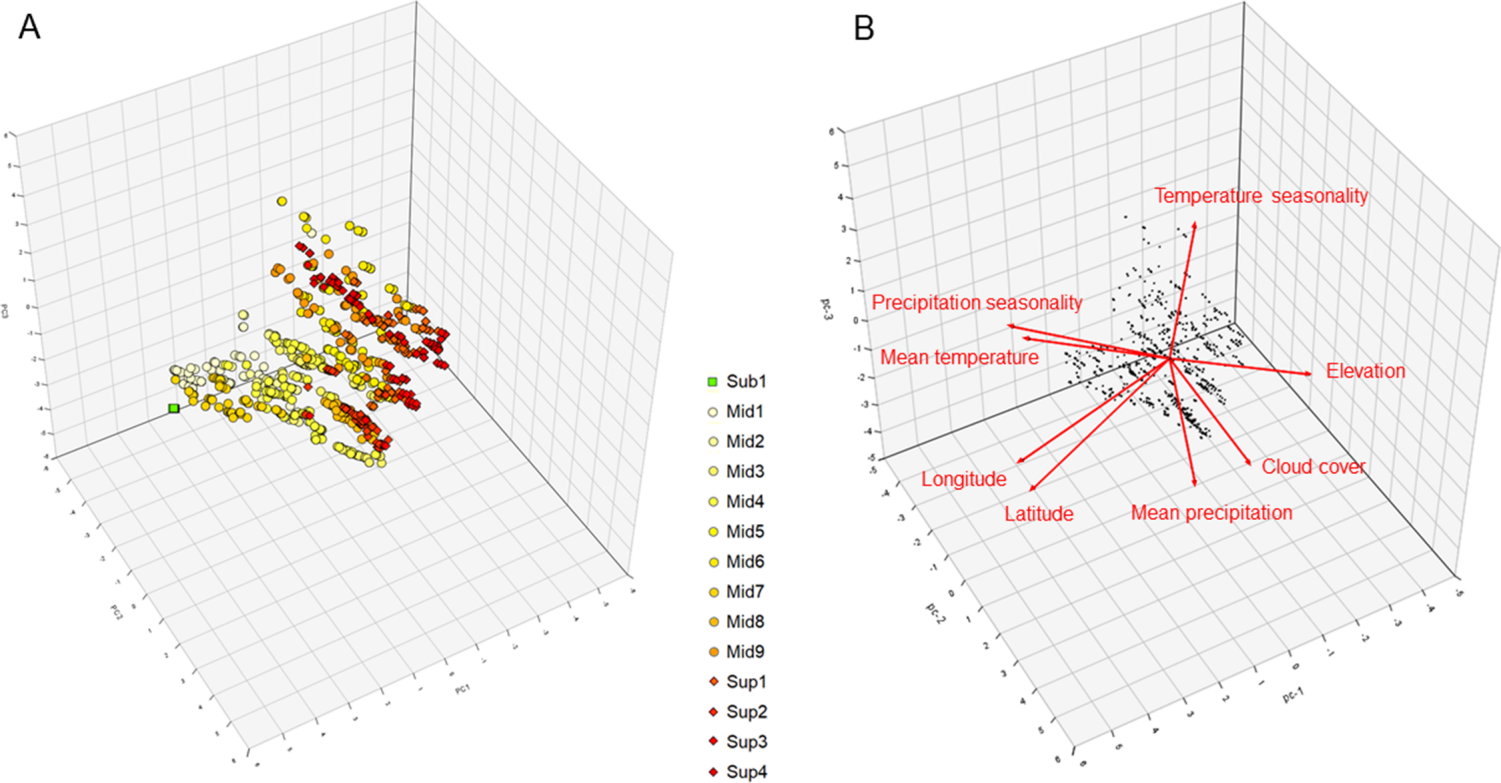
3D representation of the (A) vegetation plots and (B) geo-climatic variables along the three first principal components of the principal component analysis (cumulative variance explained 76.9%) conducted on the páramo vegetation dataset. The K-means optimal partition clusters are highlighted in symbol form and colour. Square and in green: sub-páramo; circle and in yellow: mid-páramo; diamond and in orange or red: super-páramo. The artificial cluster with no diagnostic or climatic indicator species, Sub-2, was not included in this analysis. *Sub-1: Guaramacal sub-páramo, Mid-1: Périja-Santa Marta mid-páramo, Mid-2: Eastern cordillera midpáramo, Mid-3: Central and western cordilleras mid-páramo, Mid-4: Mixed group of humid mid-páramo, Mid-5: Carchi mid-páramo, Mid-6: Ecuadorian mid-páramo, Mid-7: Venezuelan mid-páramo and lower superpáramo, Mid-8: The Nevados upper mid-páramo, Mid-9: The Ecuadorian upper mid-páramo, Sup-1: Lower humid super-páramo, Sup-2: The Nevados super-páramo, Sup-3: Upper humid super-páramo, Sup-4: Upper dry Ecuadorian super-páramo*. Red arrows show the direction and explaining power of each geo-climatic variable.

The first three components (PC) of the geo-climatic PCA represented a cumulative 76.9% of the variance. According to our results (Figs. 4A and 4B), PC1 (38.3%) was mostly a geographical axis positively marked by increasing latitude and longitude, as shown by the northern clusters Sub-1 and Mid-1, and negatively marked by increasing elevation with Sup-3 and Sup-4. PC2 (22.8%) was more of a humidity axis, positively marked by increasing precipitation and cloud cover, as shown by clusters Mid-3 and Mid-4, and negatively marked with increasing precipitation seasonality, as shown by clusters Mid-9 and Sup-4. Finally, PC3 (15.8%) was more of a temperature-related axis, positively marked by increasing annual temperature and temperature seasonality, as shown by clusters Mid-1 and Mid-6, and negatively marked by increasing elevation and decreasing latitude with clusters Sup-2 and Sup-3.

### Description of the phytogeographical units

A description of the phytogeographical units based on their floristics, i.e. diagnostic species and dominant species (for further detail, see synoptic table in Supplemental Information 4), geo-climate, and complemented by bibliographical research on ecology is given below following an elevational and North–South order.

### The sub-páramo

Two main sub-páramo clusters were identified.

*Sub-1 (38 plots)*—*Guaramacal sub-páramo*—The plots contained in this cluster were mostly distributed in the low-elevation páramos of Guaramacal (∼9°N–70°W) and punctually Táchira (∼8°N–72°W) in Venezuela. Among the diagnostic species were the small tree *Libanothamnus griffinii* (OI: 0.43) and shrub-tall *Ruilopezia lopez-palacii* (OI: 0.78) giant rosettes, bamboos including *Chusquea angustifolia* (OI: 0.78) and *Chusquea steyermarkii* (OI: 0.43), and shrubs such as *Hypericum paramitanum* (OI: 0.65). Dominant species present in most plots included the shrub *Pernettya prostrata*, tall grass *Cortaderia hapalotricha* and clubmoss *Lycopodium clavatum*. Considering such characteristics, the cluster was considered representative of the isolated and rich in endemics sub-páramo from Guaramacal dominated by mixed sub-páramo dwarf forests and shrublands with giant rosettes and bamboos (Cuello & Cleef, 2009).

*Sub-2 (190 plots)*—*Widespread sub-páramo*—This cluster was unresolved and considered artificial, because it included widespread vegetation plots that lacked floristic similarity. It was considered mostly a sub-páramo cluster due to its general low-elevation (<3,100 m), however some plots included here came from high-elevation. At this stage, no significant floristic coherence could be detected at species level, hence no valid list of diagnostic species could be provided. The dominant species, *Pernettya prostrata* and *Hesperomeles obtusifolia* were very common páramo shrubs present in respectively 22 and 20% of this cluster’s plots and also important in other clusters. Because of the cluster’s heterogeneity, its large amounts of plots and extensive species list (1,186), we conducted a complementary clustering analysis to intend revealing sub-clusters. To do so, we carried out a K-means clustering analysis on the cluster’s dataset at genus level, followed by the calculus of Ochiai Index values. In this case, the optimal partition could be identified using the silhouette statistical criterion, which showed a peak value of 0.08326 at the division into three clusters (Rousseeuw, 1987). Three main sub-clusters were identified: (1) sub-cluster 1—semi-dry grassland, (2) sub-cluster 2—shrubland and dwarf forests and (3) sub-cluster 3—secondary succession vegetation (Table 1). However, because the OI calculated at genus level were only considering this particular cluster, this analysis provided information on tendencies and not diagnostic taxa. Additional data would be required to segregate better this cluster.

**Table 1:** Results of the K-means clustering analysis and Ochiai Index (OI) calculus conducted on the *Sub-2 Widespread sub-páramo* vegetation dataset at genus level.

	*Number of plots*	*Characteristic genera (Ochiai Index value)*
***Sub-cluster 1***	56	*Carex* (0.38), *Cerastium* (0.32), *Stellaria* (0.28), *Echeveria* (0.27), *Lasiocephalus* (0.27)
***Sub-cluster 2***	46	*Weinmannia* (0.77), *Lepanthes* (0.68), *Bomarea* (0.67), *Ilex* (0.40), *Miconia* (0.66)
***Sub-cluster 3***	87	*Hypericum* (0.73), *Calamagrostis* (0.73), *Rhynchospora* (0.67), *Lycopodium* (0.65), *Paspalum* (0.65)

**Notes:**

The OI values show tendencies but are not to be interpreted numerically because of the vegetation data scale and numbers.

### The mid-páramo

Nine clusters of mid-páramo phytogeographical units spread over a large geographic gradient could be distinguished, ranging from giant rosette dominated communities in Venezuela to the North–East to mixed grasslands with giant rosettes in Colombia and tussock grasslands in Ecuador and Peru to the South–West. Altitudinal divisions between lower and upper mid-páramo were also perceived in some Ecuadorian and Colombian páramo areas.

*Mid-1 (119 plots)—Périja-Santa Marta mid-páramo*—Most plots from this cluster came from the Sierra de Périja and Sierra Nevada de Santa Marta close mountain ranges from northern Colombia. Few plots from the Colombian eastern cordillera and Venezuela that shared a similar semi-dry and seasonal climate were also included in this cluster. Its diagnostic species were the shrubs *Pentacalia albotecta* (OI: 0.41) and several *Hypericum* species, including *Hypericum magdalenicum* (OI: 0.41), *Hypericum stenopetalum* (OI: 0.36) and *Hypericum baccharoides* (OI: 0.33). Diagnostic herbs included *Ranunculus spaniophyllus* (OI: 0.38) and *Lupinus carrikeri* (OI: 0.32). Given that the tussock grass *Calamagrostis effusa* was dominant, this cluster mostly represented the mid-páramo mixed *Calamagrostis* grasslands with many locally diversified and endemic shrubs from this northern Colombian biogeographic complex (Rivera-Díaz & Fernández-Alonso, 2003; Pinto-Zárate & Rangel-Churio, 2010a).

*Mid-2 (137 plots)—Eastern cordillera mid-páramo*—This cluster was mainly distributed in the Colombian eastern cordillera. Among its diagnostic species were the dominant giant rosette *Espeletia grandiflora* (OI: 0.62), shrubs such as *Diplostephium phylicoides* (OI: 0.73) and *Arcytophyllum nitidum* (OI: 0.51), the grass *Calamagrostis bogotensis* (OI: 0.50), and herbs such as *Bartsia santolinifolia* (OI: 0.45) and *Castratella piloselloides* (OI: 0.54). The bamboo *Chusquea tessellata* and tussock grass *Calamagrostis effusa* were also dominant species, which suggested that both the mid-páramo from the drier eastern slope dominated by grasslands and the mid-páramo from the wetter western slope dominated by mixed-bamboo vegetation (Cleef, 1981) were represented in this cluster.

*Mid-3 (164 plots)—Central and western cordilleras mid-páramo*—This cluster’s plots were mostly distributed in the central and western cordilleras as well as the southern Andes in Colombia. The climatic conditions associated with this cluster informed of certain humidity, higher in the western cordillera and lower in the central cordillera. Diagnostic species included the shrubs *Diplostephium schultzii* (OI: 0.43), *Monnina revoluta* (OI: 0.32) and *Baccharis macrantha* (OI: 0.31) as well as the herbs *Niphogeton ternata* (OI: 0.40) and *Bartsia orthocarpiflora* (OI: 0.29). Among the dominant species were found the tussock grass *Calamagrostis effusa*, the giant rosette *Espeletia hartwegiana*, the shrub *Pentacalia vaccinioides* and fern *Blechnum loxense*. Considering such species composition, this cluster was therefore considered representative of the mixed grasslands with shrubs from the semi-humid and humid mid-páramo of the central and western Colombian cordilleras (Pinto-Zárate & Rangel-Churio, 2010b).

*Mid-4 (161 plots)—Mixed group of humid mid-páramo—*This cluster did not represent a fully coherent biogeographical unit and was considered artificial, because it included geographically widespread plots in Colombia and showed no significant diagnostic species, while its dominant species were common páramo plants. However, the geo-climatic PCA suggested that this cluster had a very strong humidity component, which was also sustained by the presence of common species such as the bamboo *Chusquea tessellata* and herbs like *Arcytophyllum muticum* and *Carex bonplandii* (Luteyn, 1999). An often repeated species combination within this cluster’s plots was the assemblage of the dominant tussock grass *Calamagrostis effusa* with the low shrub *Pernettya prostrata*, both widespread páramo species, and the prostrate plant *Arcytophyllum muticum*. As a result, this cluster undoubtedly represented humid mid-páramo from Colombia and showed certain floristic affinities with Mid-2 and Mid-3, but it was probably generated by grouping plots that lacked the diagnostic species of the other clusters but could not create a new valid one at this stage.

*Mid-5 (55 plots)—Carchi mid-páramo*—The plots included in this cluster came from the Ecuador-Colombia Andean border. Among the diagnostic species encountered were the local giant rosette *Espeletia pycnophylla* (OI: 0.90), shrubs such as *Brachyotum lindenii* (OI: 0.52) and *Diplostephium rhododendroides* (OI: 0.75), as well as the herbs *Chaptalia cordata* (OI: 0.40) and *Lupinus pubescens* (OI: 0.66). Consequently, and considering the dominance of the tussock grass *Calamagrostis effusa,* this cluster was revealed as the particular mid-páramo of mixed grasslands with the only *Espeletia* giant rosette species known to Ecuador (Moscol-Olivera & Cleef, 2009), and where transitionally occurs the southern-ending distribution for both *Espeletia* spp. and *Calamagrostis effusa*.

*Mid-6 (139 plots)—Ecuadorian mid-páramo*—This cluster’s plots were mostly located in Ecuador and to a lesser extent in Peru, and showed a certain warm and relatively seasonal climatic character. Diagnostic species for this cluster were mostly herbs, including *Ranunculus peruvianus* (OI: 0.31), *Carex pygmaea* (OI: 0.32), *Galium corymbosum* (OI: 0.40), *Dorobaea pimpinellifolia* (OI: 0.23) and *Senecio chionogeton* (OI: 0.35). Among the most dominant species was *Calamagrostis intermedia*, which confirmed this cluster’s representation of the common semi-dry grasslands mixed with shrubs of *Pentacalia* spp., *Diplostephium* spp. and *Monnina* spp. (Ramsay, 1992) of the Ecuadorian and Peruvian mid-páramo.

*Mid-7 (87 plots)—Venezuelan mid-páramo and lower super-páramo*—These plots were distributed in most Venezuelan páramos, especially in the Cordillera de Mérida (∼8°N–71°W), and covered a wide altitudinal range over the mid-páramo and lower super-páramo altitudinal belts. Among the most important diagnostic species were the giant rosette *Espeletia schultzii* (OI: 0.73), shrubs such as *Baccharis prunifolia* (OI: 0.44) and *Oxylobus glanduliferus* (OI: 0.44) and herbs like *Azorella julianii* (OI: 0.30) and *Poa petrosa* (OI: 0.48). In addition, the shrub *Hypericum laricifolium* and prostrate herb *Acaena cylindrostachya* were common. The lack of tussock grass dominance in this cluster contrasted to the other mid-páramo clusters. It therefore represented the dominant semi-dry páramos of Venezuela, where the diversified giant rosettes *Espeletia* spp., *Coespeletia* spp. and *Ruilopezia* spp., co-occur with shrubs such as *Baccharis* spp. and *Chaetolepis* spp. (Monasterio & Reyes, 1980; Diazgranados, 2012). However, the lower-super-páramo from Venezuela was also represented in this cluster as shown by the diagnostic herbs *Hinterhubera imbricata* (OI: 0.50) and *Draba pulvinata* (OI: 0.36) of high-elevation deserts (Berg, 1998).

*Mid-8* (72 plots)—*The Nevados upper mid-páramo*—The plots from this cluster were mostly distributed in the Nevados páramo (∼4.8°N–75.3°W) and punctually in the Sumapaz páramo (∼4°N–74.2°W) in Colombia around 4,000 m elevation. This distinctive cluster was characterised by the diagnostic tussock grass *Calamagrostis recta* (OI: 0.69), shrubs such as *Pentacalia vernicosa* (OI: 0.44) and *Baccharis rupicola* (OI: 0.39), and herbs including *Gentianella dasyantha* (OI: 0.42) and *Aa colombiana* (OI: 0.29). Among the dominant species were the giant rosette *Espeletia hartwegiana* and herbs such as the common *Oreomyrrhis andicola* and *Hypochaeris sessiliflora*. With such floristic characteristics, this cluster was therefore distinguished from the common mixed grassland of *Calamagrostis effusa* and *Espeletia* spp. of the Colombian mid-páramo (Salamanca, Cleef & Rangel-Churio, 2003), and instead represented the ecotone between mid-páramo and super-páramo in these selected mountain ranges.

*Mid-9 (93 plots)—The Ecuadorian upper mid-páramo*—The plots contained in this cluster were distributed in Ecuador around 4,000 m in relatively seasonal páramos. Among the diagnostic species were the grasses *Calamagrostis fibrovaginata* (OI: 0.33) and *Festuca andicola* (OI: 0.47), as well as the herbs *Gentianella cerastioides* (OI: 0.55), *Cerastium imbricatum* (OI: 0.42) and the acaulescent rosette *Valeriana rigida* (OI: 0.35). Common species included other grasses, in particular *Calamagrostis intermedia*, and cushion forming plants, mostly from the genus *Azorella*, such as *Azorella pedunculata* and *Azorella aretioides*. This cluster therefore represented the upper mid-páramo transition from Ecuador dominated by mixed grasslands with cushion plants (Ramsay, 1992).

### The super-páramo

Four clusters of Colombian and Ecuadorian super-páramos were revealed.

*Sup-1 (156 plots)—Lower humid super-páramo*—The plots included in this cluster were distributed in relatively humid environments around 4,200 m in Ecuador and southern Colombia. Among the diagnostic species were the shrub *Diplostephium rupestre* (OI: 0.50), grasses such as *Festuca asplundii* (OI: 0.46) and *Calamagrostis ecuadoriensis* (OI: 0.35), and herbs including *Gentianella nummulariifolia* (OI: 0.39) and *Valeriana bracteata* (OI: 0.36). Commonly found species included cushion plants such as *Xenophyllum humile, Azorella aretioides* and *Plantago rigida*. Therefore, this cluster representated of the transitional cushion plant communities with small shrubs from the lower-sub-páramo of semi-humid and humid mountains in Ecuador and southern Colombia. In contrast to the Mid-9 cluster, which contained mix grass-cushion communities dominated by grasses of the lower ecotone, Sup-1 represented the cushion-dominated vegetation of the upper ecotone where environmental humidity is constant, soils are deep and frost is limited (Sklenář, 2009). This zonal cluster resembled azonal bogs and mire vegetation due to shared dominant species, but its accompanying diagnostic species were key to differentiate them.

*Sup-2 (81 plots)—The Nevados super-páramo*—Most of the plots included here came from the Nevados páramo and secondarily from the Sumapaz páramo. The grass *Bromus lanatus* and the small herbs *Hypochaeries sessiliflora* and *Valeriana pilosa* were dominant species. Among the diagnostic species were the small shrubs *Loricaria colombiana* (OI: 0.20) and *Pentacalia gelida* (OI: 0.47), the grass *Agrostis araucana* (OI: 0.44) and several herbs including *Lupinus alopecuroides* (OI: 0.36), *Erigeron chionophilus* (OI: 0.57), *Senecio isabelis* (OI: 0.34) and *Draba pennell-hazenii* (OI: 0.27). This cluster therefore represented the super-páramo from the Nevados páramo, including the lower super-páramo, with *Loricaria* shrublands and blue meadows, and upper super-páramo, characterised by high-elevations deserts (Salamanca, Cleef & Rangel-Churio, 2003).

*Sup-3 (94 plots)—Upper humid super-páramo*—This cluster was mainly distributed around 4,400 m in central and northern Ecuador, and also in the Nevados páramo. Diagnostic species included the herbs *Senecio nivalis* (OI: 0.70), *Erigeron ecuadoriensis* (OI: 0.46) and *Draba aretioides* (OI: 0.43), as well as two indicators of certain humidity, the grass *Calamagrostis ligulata* (OI: 0.45) and the prostrate herb *Ourisia muscosa* (OI: 0.40). Other associated common species were *Agrostis foliata, Xenophyllum humile, Cerastium floccosum* and *Luzula racemosa*. As a result, this cluster represented the cold and semi-humid to humid upper-super-páramo found mostly in Ecuador. At this elevation, climatic conditions usually are very drastic with permanent night frost and high solifluction that confines the vegetation to few available microsites, making it almost desertic. In the case of humid upper super-páramo, the vegetation is organised in small patches with an overall ground-cover of 15–20%, which contrasts with dry upper super-páramos (Sklenář, 2000).

*Sup-4 (61 plots)—Upper dry Ecuadorian super-páramo*—The plots included here are restricted to the dry inter-Andean valley slopes of Ecuador, at elevations around 4,500 m. The grasses *Calamagrostis mollis* (OI: 0.49) and *Festuca vaginalis* (OI: 0.39) as well as small herbs such as *Astragalus geminiflorus* (OI: 0.72), *Nototriche jamesonii* (OI: 0.59) and *Draba depressa* (OI: 0.50) were diagnostic. Other common species were the basal rosette *Hypochaeris sessiliflora* and prostrate plant *Baccharis caespitosa*. This cluster represented the dry upper super-páramo, including rain-shadow deserts (e.g., the Chimborazo mountain) in Ecuador, which contrarily to its humid counterparts (Sup-3), shows 10–15% vegetation ground cover and includes highly specialised and endemic species (Sklenář, 2000).

### Similarities between phytogeographical units

**Figure 5:**
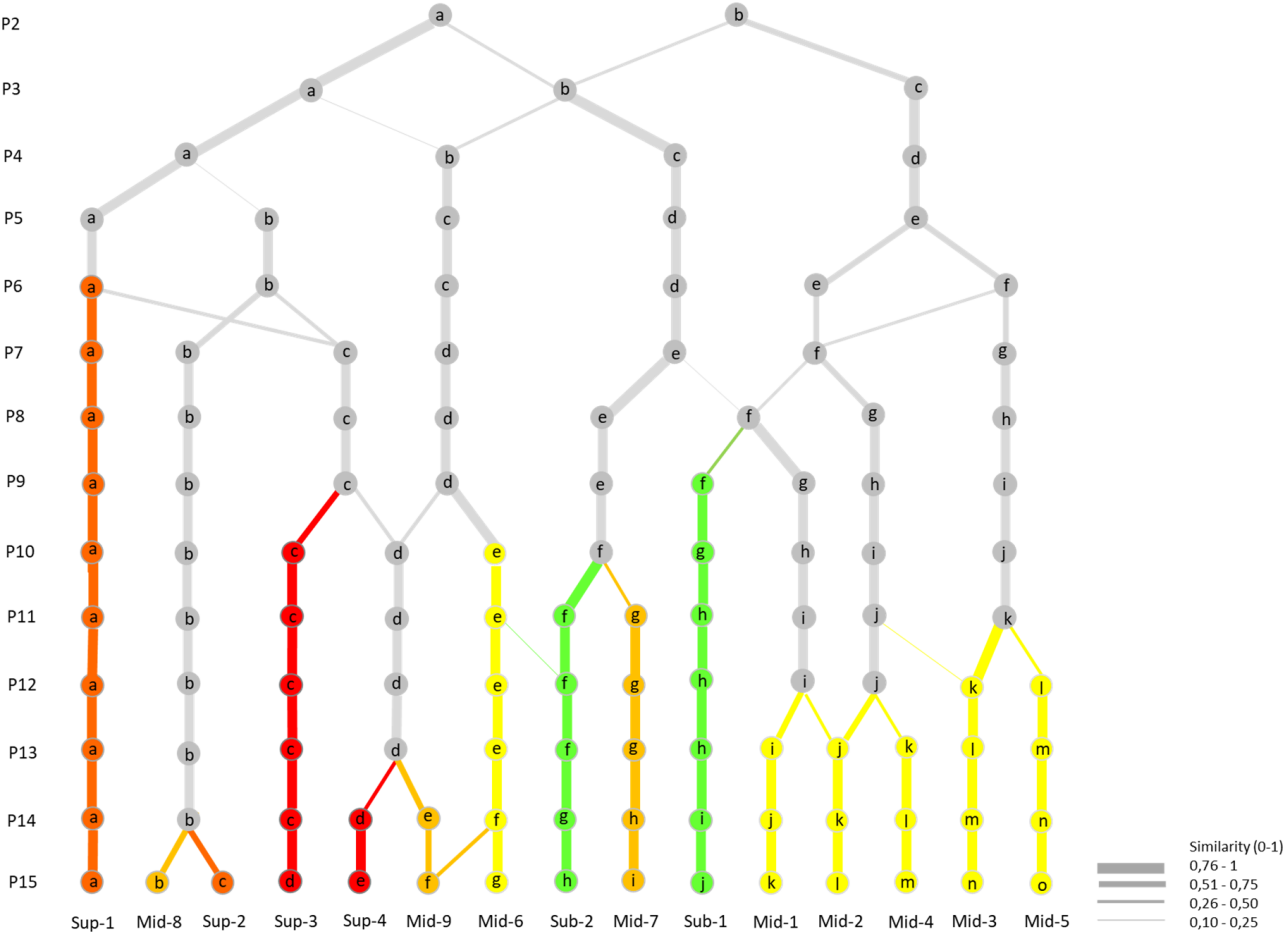
Hierarchical classification of the K-means successive partitions up to the optimal partition of 15 clusters, based on plot content similarities (0–1). Colour arrows show the appearance and maintaining of clusters through partitions without major plot content modifications (<0.5). In green: sub-páramo; in light yellow: mid-páramo; in dark yellow: upper mid-páramo; in orange: lower super-páramo; in red: upper super-páramo. *Sub-1: Guaramacal sub-páramo, Sub-2: Widespread sub-páramo, Mid-1: Périja-Santa Marta mid-páramo, Mid-2: Eastern cordillera midpáramo, Mid-3: Central and western cordilleras mid-páramo, Mid-4: Mixed group of humid mid-páramo, Mid-5: Carchi mid-páramo, Mid-6: Ecuadorian mid-páramo, Mid-7: Venezuelan mid-páramo and lower superpáramo, Mid-8: The Nevados upper mid-páramo, Mid-9: The Ecuadorian upper mid-páramo, Sup-1: Lower humid super-páramo, Sup-2: The Nevados super-páramo, Sup-3: Upper humid super-páramo, Sup-4: Upper dry Ecuadorian super-páramo.*

The results of the transformation of the K-means clustering into a hierarchical classification are shown in Fig. 5 and described below following the increasing order of division observed. Already at the division into four clusters, the dataset broadly divided latitudinally into four main units: one Ecuadorian mid-páramo unit (P4-b), one sub-páramo/Venezuelan mid-páramo unit (P4-c), one Colombian mid-páramo unit (P4-d), and one altitudinally discriminated unit, the super-páramo unit (P4-a). In further divisions, the Ecuadorian mid-páramo and super-páramo branches continued to interchange plots, as did the sub-páramo/Venezuelan mid-páramo and Colombian mid-páramo branches, but they remained mostly separated from one another.

First, early at the fifth clusters partition, the super-páramo group (P4-a) divided, first separating the transitional lower super-páramo from Ecuador (P5-a) from the rest, and then at seventh clusters partition, isolating the Nevados high-elevation páramo (P7-b) from the Ecuadorian high elevation super-páramos (P7-c). The latter group redivided at the 10th clusters partition by combining plots with the Ecuadorian mid-páramo cluster (P9-d) to create the humid upper super-páramo cluster (P10-d) and isolate the dry upper super-páramo cluster (P10-c). At the 15th clusters partition, the Nevados cluster P7-b divided by elevation into Mid-8, which was a transitional páramo/super-páramo ecotone, from its directly above super-páramo cluster Sup-2.

Second, the Ecuadorian mid-páramo group (P4-b) separated first at the 10th clusters partition based on elevation between lower mid-páramo (P10-e) and humid upper mid-páramo (P10-d). This latter cluster parted later at the 14th clusters partition, and divided by elevation into upper mid-páramo (P14-e) and upper dry super-páramo (P14-d).

Third, the Colombian mid-páramo group (P4-d) separated quickly at the sixth clusters partition into western (P6-f) and eastern clusters (P6-e). The eastern cluster divided progressively between the eighth and 13th clusters partitions, isolating clusters of the Colombian eastern cordillera (P13-j), the Santa Marta/Périja complex (P13-i) and the mixed humid Colombian grasslands (P13-k). The western cluster divided later at the 12th clusters partition into the Carchi mid-páramo cluster (P12-l) and the Colombian central and western cordilleras cluster (P12-k).

Lastly, the sub-páramo/Venezuelan mid-páramo group (P4-c) divided at the eighth clusters partition into a mixed cluster with the Colombian mid-páramo group, which eventually lead to the particular Guaramacal sub-páramo cluster (P9-f). Later, at the 12th clusters partition, the other group divided into the general sub-páramo cluster (P11-f) and Venezuelan páramo cluster (P11-g).

## Discussion

This study is the first phytogeographical regionalisation of the páramo which, based on a substantial dataset of biological data with a wide distribution, revealed strong floristic and geographic divisions throughout the biogeographical province. Our clustering analyses identified 15 clusters, 13 of which were natural clusters comparable to phytogeographical units spread over latitudinal and altitudinal gradients. Nonetheless, two clusters resulted artificial at this stage of division, (1) Sub-2, which included plots of underrepresented vegetation that did not fit into other clusters, and (2) Mid-4, which in addition had shared humidity indicator species. These two clusters would probably divide into floristically meaningful sub-clusters in a more detailed K-means partition, however because of our broad-scale biogeographical focus, a better phytoregionalisation of the páramo could only be obtained by considering these underrepresented vegetation types and increasing plot numbers. Regarding the natural clusters, we stress the importance to focus additional scientific and conservation research on Sub-1, Sup-1 and Sup-3 which, by separating early on the hierarchical classification and presenting many diagnostic species with high Ochiai Index values, emerged as particularly relevant phytogeographical units with possibly highly biodiverse and endemic flora.

The sub-páramo is usually considered the most biodiverse páramo altitudinal belt in terms of species richness and plant communities, because it shares species with the adjacent Andean forests and shows the highest topographical and environmental heterogeneity (Rangel-Churio, 2000; Stein, Gerstner & Kreft, 2014; Llambí, 2015; Peyre, 2015). Our analyses had difficulties separating sub-páramo phytogeographical units, and only the Guaramacal sub-páramo stood out, thanks to its unique flora, high endemism, isolated situation (Cuello & Cleef, 2009) and good data representation. By contrast, most other sub-páramo vegetation plots were included into the artificial Sub-2 cluster. There could be different non-exclusive explanations for this unexpected finding, for example: (1) the under-representation of plot data for this often disregarded ecotonal altitudinal belt, and (2) that niche differentiation in the sub-páramo would be less pronounced than in the more isolated and environmentally constrained mid-páramo and particularly super-páramo, which would difficult the segregation of valid units. Under this second perspective, it might be useful to focus on other potentially significant drivers of species assembly processes such as functional and phylogenetic diversity to differentiate the sub-páramo phytogeographical units (Pavoine & Bonsall, 2011; Chalmandrier et al., 2015). When dividing Sub-2 into three sub-clusters based on a genus-level clustering, and after separating the mixed secondary vegetation and semi-dry grassland plots (sub-clusters 1 and 3), we obtained a better defined sub-páramo cluster (sub-cluster 2). Even though any interpretation of this cluster would be incomplete due to the taxonomic level used, we identified indicators of the common sub-páramo dwarf forests including the general tree and shrub genera *Miconia*, *Weinmannia* and *Ilex*, the climber *Bomarea* and orchid *Lepanthes* among others (Luteyn, 1999; Rangel-Churio, 2000). Thanks to this promising preliminary sub-páramo cluster, we believe that by adding new vegetation data from the low-elevation páramo areas, additional true sub-páramo phytogeographical units could be identified and characterised. Finally, we observed in the hierarchical classification results that the sub-páramo clusters shared more resemblance with the Venezuelan and Colombian mid-páramo clusters rather than the Ecuadorian mid-páramo clusters. We think this finding might be indicator of, (1) a more gradual vegetation transition between sub-páramo and mid-páramo in Venezuela and Colombia, revealing perhaps less human intervention at this ecotone compared to the southern páramos, (2) simply a higher shrub component in the Venezuelan and Colombian mid-páramos (Rangel-Churio, 2000) or (3) a bias of preferential sampling of sub-páramo vegetation in the northern páramos. The sub-páramo is very threatened in general by the intensification of agriculture and pasture that induce the retraction of dwarf forests and shrublands for the benefit of crop and grassland expansion (Molinillo & Monasterio, 2002; Llambí, 2015). Nonetheless, some sub-páramos located in remote and difficult-to-access areas, in particular in eastern Venezuela, southern Ecuador and Peru, have remained relatively pristine to date (Weigend, 2002; Lozano, Cleef & Bussmann, 2009) and therefore require urgent scientific efforts to better understand their ecology and biogeography, but also to promote their conservation.

In the mid-páramo belt, dominant plant species are often also diagnostic, which helps identify vegetation types (Sklenář & Ramsay, 2001), and at broader-scale phytogeographical units. The Colombian páramos are typically humid, principally thanks to the *Inter-tropical convergence zone,* while the Ecuadorian–Peruvian and Venezuelan páramos are under a stronger influence from the drier Humboldt current and North–East trade winds respectively (Luteyn, 1999; Martínez et al., 2011). Our clustering results illustrated this broad-scale climatic pattern for the mid-páramo, with a gradient going from grass-dominated biogeographical units in Peru and Ecuador, to more humid mixed grass, giant-rosette and bamboo units in Colombia and to drier giant rosette-dominated units in Venezuela (Monasterio & Reyes, 1980). In addition, the hierarchical classification also emphasized this gradient, with the dominance of (1) *Calamagrostis effusa* and *Calamagrostis intermedia* species differentiating the northern Colombian and southern Ecuadorian domains respectively, and (2) *Espeletia* species dividing the northern domain into smaller phytogeographical units, for instance in Colombia with *Espeletia grandiflora* in the eastern cordillera and *Espeletia hartwegiana* in the western (subsp. *hartwegiana*) and central cordilleras (subsp. c*entroandina*) (Rangel-Churio, 2000; Pinto-Zárate & Rangel-Churio, 2010b). This study rejoined previous findings based on páramo floristic data in Colombia, which differentiated northern, eastern, central-southern and western sectors (Londono, Cleef & Madriñán, 2014). Nonetheless, classifying the mid-páramo in Colombia is particularly challenging, due to the high abundance of bamboos, often the species *Chusquea tessellata*, which is an indicator of humidity that tends to outweigh other biogeographical characteristics, as seen in the artificial Mid-4 cluster. Moreover, proportions of these floristic elements vary between and also within the cordilleras, essentially between the eastern and western slopes, for example comparing the drier inter-Andean valleys with the wetter Amazonian slope (Cleef, 1981; Rangel-Churio, 2015). Finally, the Venezuelan páramo was identified in our analyses as a particular unit from a floristic point of view. It was primarily characterised by the diversified Espeletiinae giant rosettes (Diazgranados, 2012; Diazgranados & Barber, 2017), separated early from the other clusters, and showed many diagnostic species with high OI values. Therefore, and even though our analyses did not segregate by elevation the Venezuelan páramo at this stage, the resulting findings support previous studies that distinguished these páramos from the central and southern páramos based on flora (Cuesta et al., 2017). Because the mid-páramo belt is mostly under human influence, either fragmented or homogenised by anthropogenic activities (Molinillo & Monasterio, 2002), it would be useful to correlate our results with a broad-scale páramo land-use model, so to better understand which páramo phytogeographical units are more natural or anthropogenised, hence to guide and prioritise conservation efforts.

The super-páramo belt is not continuously distributed throughout the páramo province, but instead situated as biogeographical continental islands, characterised by constraining edaphic and climatic conditions that result in high niche differentiation, biota specialisation and endemism (Luteyn, 1999; Anthelme et al., 2014). Our results identified several geographically and environmentally distinct phytogeographical units in Ecuador and Colombia, but could not represent well the more scarcely sampled super-páramo areas of Venezuela and to some degree northern Colombia. Because of the super-páramo’s insularity, its flora is highly endemic and organised as a complex vegetation with narrow distribution and strong ecological network and interactions (Sklenář & Balslev, 2005). In general, the lower humid super-páramo, which is located in the *Humid Upper Condensation Belt* and corresponds to relatively continuous low shrublands with or without cushions, was well differentiated from the desertic upper super-páramo where the very stressful environmental conditions determine plants’ survival, growth and reproduction (Cleef, 1981; Sklenář & Ramsay, 2001). In turn, the upper super-páramo was divided into drier and more humid super-páramos, as clearly seen in Ecuador (Sklenář, 2000). No such clear separation could be observed for the Colombian super-páramo, but an interesting cluster, Sup-2, mostly containing plots from the Nevados páramo but also from Sumapaz, could be distinguished. The remaining Colombian super-páramos were unfortunately spread over different clusters and might have been overlooked because of low data representativeness. Finally, the Venezuelan super-páramo could not be well differentiated and remained included with the general mid-páramo Venezuelan cluster Mid-7, which might be due in part to the lacking upper condensation belt specific vegetation associated to the drier climate (Monasterio & Reyes, 1980; Berg, 1998). The super-páramo contains the highest elevation plants in the northern Andes, which have mostly escaped land-use expansion and intensification so far, thanks to the poverty of the soils and harsh climates (Luteyn, 1999; Sklenář, 2000). However, the imminent climate change and its associated anthropogenic change should threaten the super-páramo in the near future, and it is therefore crucial to understand better the long debated and mostly unknown adaptation and migration capacity of these plants and ecosystems under these new environments (Lenoir et al., 2008; Morueta-Holme et al., 2016; Delnevo et al., 2018, Graae et al., 2018).

## Conclusion

The Andean páramo is a widely distributed biogeographical province, a true biodiversity hotspot and the perfect model to study tropical alpine ecosystems worldwide (Sklenář, Hedberg & Cleef, 2014). Our study is first to bioregionalise the páramo based on a substantial vegetation dataset and describe its main phytogeographical units spread over almost 20° latitude and 2,000 m elevation. A total of 15 biogeographical units were identified, 13 of which were considered natural, and distributed as follows: two representing the sub-páramo, nine the mid-páramo and four the super-páramo. The phytoregionalisation of the páramo was considered robust and showed good floristic differentiation along geographic and environmental gradients. We believe our study provides novel insight on páramo biogeography, offers a strong base for future ecological and biodiversity management studies and contributes to slowly filling the knowledge gap on tropical alpine research (Anthelme & Lavergne, 2018).

Among the limitations encountered was the data coverage of the páramo province which, even though substantial and geographically well spread, was not sufficient to represent all ecosystems, which led to inconsistent clusters such as Sub-2 or Mid-4. This is a common issue in bioregionalisations over broad-scales (Kreft & Jetz, 2010; Andrés & Font, 2011), and potential solutions would include (1) increasing the sampling effort in under-studied ecosystems (e.g., sub-páramo and super-páramo) and areas (e.g., Peruvian and Colombian páramos from the central and oriental cordilleras), and (2) carrying out additional resampling based on ecosystems. It would also be relevant to include further environmental and land-use data in the future to complement the analyses and strengthen the socio-ecological interpretations of the páramo bioregionalisation. Finally, it would be interesting to add to our results, data from other closely related ecosystems to the páramo, for example the humid Puna, Bolivian Yungas, Amazonian volcanoes and Central American páramo to increase the study-scale and complete the phytogeographical regionalisation of humid high elevation ecosystems in the Neotropics.

## Supplemental Information

10.7717/peerj.4786/supp-1 Supplemental Information 1 Five seed plots for each phytogeographical unit of the optimal partition (15 clusters) to conduct a semi-supervised non-hierarchical classification of the VegPáramo dataset and reproduce our analyses.

10.7717/peerj.4786/supp-2 Supplemental Information 2 Climatic conditions across the páramo biogeographical province. From left to right: annual temperature, temperature seasonality, annual precipitation, precipitation seasonality, cloud cover. All variables were obtained from the CHELSA 1.2 project.

10.7717/peerj.4786/supp-3 Supplemental Information 3 Characteristics of the outlier plots not considered in Figs. 2 and 3.

10.7717/peerj.4786/supp-4 Supplemental Information 4 Synoptic table for the optimal partition (15 clusters) of the non-hierarchical clustering analysis on the VegPáramo data. Percentage of presence (> 1%) of each species within each cluster, corresponding to a phytogeographical unit. *Sub-1: Guaramacal sub-páramo, Sub-2: Widespread sub-páramo, Mid-1: Périja-Santa Marta mid-páramo, Mid-2: Eastern cordillera mid-páramo, Mid-3: Central and western cordilleras mid-páramo, Mid-4: Mixed group of humid mid-páramo , Mid-5: Carchi mid-páramo, Mid-6: Ecuadorian mid-páramo, Mid-7: Venezuelan mid-páramo and lower super-páramo, Mid-8: The Nevados upper mid-páramo, Mid-9: The Ecuadorianuppermid-páramo, Sup-1: Lower humid super-páramo, Sup-2: The Nevados super-páramo, Sup-3: Upper humid super-páramo, Sup-4: Upper dry Ecuadorian super-páramo.*.

## Additional Information and Declarations

### Data Availability

The following information was supplied regarding data availability: Vegpáramo database: http://161.116.68.78/VegParamo/
