# Supplementary material for: Phytoregionalisation of the Andean páramo
Source: PeerJ. 2018 Jun 1;6:e4786. doi: 10.7717/peerj.4786 (PMC5985761; doi:10.7717/peerj.4786)
Supplement: Supplemental Information 1 [file peerj-06-4786-s001.pdf]

Supplemental information SI. 1 to Peyre et al. Phytoregionalisation of the Andean páramo

Sub-1: Guaramacal sub-páramo

Code VegPáramo	*UTM coord. (1Km)*	*Elevation (m)*	*Locality*	*Province*	*Country*
A-P00188	19PCL6920	3030	norte de Las Antenas, Ramal de Guaramacal	Trujillo	VE
A-P00196	19PCL6922	2860	norte de Las Antenas, Ramal de Guaramacal	Trujillo	VE
A-P00200	19PCL6921	2980	norte de Las Antenas, Ramal de Guaramacal	Trujillo	VE
A-P00247	19PCL6920	3040	norte de Las Antenas, Ramal de Guaramacal	Trujillo	VE
A-P00272	19PCL6921	2960	norte de Las Antenas, Ramal de Guaramacal	Trujillo	VE

Sub-2: Widespread sub-páramo

Code VegPáramo	*UTM coord. (1Km)*	*Elevation (m)*	*Locality*	*Province*	*Country*
A-P00410	18NUN8013	3550	Puente Largo, páramo de Frontino	Antioquía	CO
A-P00959	17MQR1557	3000	Reserva Biológica San Francisco	Zamora-Chinchipe	EC
A-P02718	19PAJ7287	3225	Páramo las Rosas	Táchira	VE
A-P02800	17MPQ6309	3259	Cuello del Inca	Piura	PE
A-P02852	17MPP9080	3108	Paramo de Palambe	Cajamarca	PE

Mid-1: Périja-Santa Marta mid-páramo

Code VegPáramo	UTM coord. (1Km)	Elevation (m)	Locality	*Province*	*Country*
A-P00044	18PXS5179	3850	Sierra Nevada de Santa Marta, costado Sur	Magdalena	CO
A-P00633	18PYS2843	3350	Páramo El Avión, montaña de Perijá	César	CO
A-P00672	18PYS2534	3096	sector Sabana Rubia, montaña de Perijá	César	CO
A-P01854	18PXT2414	3100	Cuchilla Guinue, Páramo de Santa Marta	Magdalena	CO
A-P01860	18PXT2206	4100	Cuchilla La Cimarrona, Páramo de Santa Marta	Magdalena	CO

Mid-2: Eastern cordillera mid-páramo

Code VegPáramo	*UTM coord. (1Km)*	*Elevation (m)*	*Locality*	*Province*	*Country*
A-P00148	18NXL0407	3150	Páramo de Monserrate, Districto Capital	Cundinamarca	CO
A-P00531	18NXK3997	3300	Parque Nacional Chingaza, hacia Alto el Gorro	Cundinamarca	CO
A-P00876	18NYM1365	3820	Páramo La Rusia, Laguna Negra, Peña Blanca	Boyacá	CO
A-P01914	18NWK7947	3550	Cuchilla la Rabona, Macizo de Sumapaz	Cundinamarca	CO
A-P01973	18NXL0701	3500	Alto del Buitre, Páramo de Cruz Verde	Cundinamarca	CO

Mid-3: Central and western cordilleras mid-páramo

Code VegPáramo	*UTM coord. (1Km)*	*Elevation (m)*	*Locality*	*Province*	*Country*
A-P00352	18NUN7715	3650	Llano Grande, páramo de Frontino	Antioquía	CO
A-P00470	18NSG8240	3630	Cumbal	Nariño	CO
A-P00620	18NUL8165	3300	Encanto, páramo de Tatamá	Chocó	CO
A-P00739	18NUL8264	3585	cerro Tamaná, vertiente E	Risaralda	CO
A-P01843	18NUH4459	3780	Pilimbalá, camino al Volcán Puracé	Cauca	CO

Mid-4: Mixed group of humid mid-páramo

Code VegPáramo	*UTM coord. (1Km)*	*Elevation (m)*	*Locality*	*Province*	*Country*
A-P00402	18NUN7912	3500	Alto del Burro, páramo de Frontino	Antioquía	CO
A-P00558	18NXL3021	3600	Parque Nacional Natural Chingaza, Palacio	Cundinamarca	CO
A-P00605	18NUL8163	3500	Reposo, páramo de Tatamá	Risaralda	CO
A-P00857	18NWK9337	3420	Páramo de Sumapaz, Laguna La Guitarra	Meta	CO
A-P00873	18NXL1284	3605	Páramos NW de Neusa,La Guargua	Cundinamarca	CO

Mid-5: Carchi mid-páramo

Code VegPáramo	*UTM coord. (1Km)*	*Elevation (m)*	*Locality*	*Province*	*Country*
A-P00279	18NSF7975	3750	Páramo El Angel	Carchi	EC
A-P00299	18NSF7872	3570	Páramo El Angel, Estación Los Encinos	Carchi	EC
A-P00329	18NTF0366	3900	Páramo Guandera, Parroquía Mariscal Sur	Carchi	EC
A-P00471	18NSG8240	3600	Cumbal	Nariño	CO
A-P02580	18NSF6778	3628	Páramo El Angel, Polylepis Lodge	Carchi	EC

Mid-6: Ecuadorian mid-páramo

Code VegPáramo	*UTM coord. (1Km)*	*Elevation (m)*	*Locality*	*Province*	*Country*
A-P01019	17MQR1458	2730	Reserva Biológica San Francisco	Zamora-Chinchipe	EC
A-P02283	17MQS0381	3600	Parque Nacional Cajas	Azuay	EC
A-P02425	17MQU8480	4000	Ladera Este, Volcán El Altar	Chimborazo	EC
A-P02633	17MQV5827	3787	ladera S del Cerro Illiniza Sur	Cotopaxi	EC
A-P02823	17MPQ7475	3468	Paramo de Espindola	Piura	PE

Mid-7: Venezuelan mid-páramo and lower super-páramo

Code VegPáramo	*UTM coord. (1Km)*	*Elevation (m)*	*Locality*	*Province*	*Country*
A-P01584	19PBK7144	4000	Páramo Media Luna, Sierra Nevada de Mérida	Merida	VE
A-P02706	19PBK9681	4328	Paramo de Piedras Blancas	Mérida	VE
A-P02726	19PBK7468	3296	Páramo La Culata	Mérida	VE
A-P02754	19PCK2497	3692	paramo de Tuñame	Trujillo	VE
A-P02774	19PBK7750	3310	Sierra Nevada de Merida, Laguna Coromoto	Mérida	VE

Mid-8: The Nevados upper mid-páramo

**Code VegPáramo**	**UTM coord. (1Km)**	**Elevation (m)**	**Locality**	**Province**	**Country**
A-P00031	18NVL6133	4150	Laguna de los Verdes, Nevado El Cisne	Caldas	CO
A-P01740	18NVL6245	4080	valle Gualí, Nevado del Ruiz	Tolima	CO
A-P02088	18NVL5636	3850	Nevado de Santa Isabel, Macizo Ruiz Tolima	Tolima	CO
A-P02132	18NVL5619	4120	Nevado del Quindio, Macizo Ruiz Tolima	Quindio	CO
A-P02140	18NVL5328	3920	Laguna Otún, Macizo Ruiz Tolima	Risaralda	CO

Mid-9: The upper Ecuadorian mid-páramo

Code VegPáramo	*UTM coord. (1Km)*	*Elevation (m)*	*Locality*	*Province*	*Country*
A-P01081	17MRV3299	4200	Ladera SW del Nevado Cayambe	Pichincha	EC
A-P01408	17MQV8429	4000	Ladera Norte del Cotopaxi, El Refugio	Pichincha	EC
A-P02607	17MQV7280	4076	ladera Este del Pichincha	Pichincha	EC
A-P02621	17MPS9692	3982	Parque Nacional El Cajas	Azuay	EC
A-P02691	17MQV8033	3999	sendero ladera SE del Cerro Rumiñahui	Pichincha	EC

Sup-1: Lower humid super-páramo

Code VegPáramo	*UTM coord. (1Km)*	*Elevation (m)*	*Locality*	*Province*	*Country*
A-P00419	18NTG3834	4200	Galeras	Nariño	CO
A-P01199	17MRU0163	4300	Laderas W-SW del Cerro Hermoso	Tungurahua	EC
A-P02420	17MQU8370	4200	Ladera Este, Volcán El Altar	Chimborazo	EC
A-P02588	17MRV1164	4230	Páramo de Papallacta	Pichincha	EC
A-P02665	17MQU6334	4245	Cerro Igualita	Tungurahua	EC

Sup-2: The Nevados super-páramo

Code VegPáramo	*UTM coord. (1Km)*	*Elevation (m)*	*Locality*	*Province*	*Country*
A-P01673	18NVL6244	4240	Nevado del Ruiz, Macizo Ruiz-Tolima	Caldas	CO
A-P01935	18NWK9835	4170	Cerro Nevado, Macizo de Sumapaz	Meta	CO
A-P02092	18NVL5834	4340	Nevado de Santa Isabel, Macizo Ruiz Tolima	Tolima	CO
A-P02114	18NVL4930	4400	Nevado de Santa Rosa, Macizo Ruiz Tolima	Risaralda	CO
A-P02180	18NVL4828	4375	Laguna Otún, Macizo Ruiz Tolima	Risaralda	CO

Sup-3: Upper humid super-páramo

Code VegPáramo	*UTM coord. (1Km)*	*Elevation (m)*	*Locality*	*Province*	*Country*
A-P01128	17MRV2246	4300	Ladera E del Antisana	Napo	EC
A-P01203	18NSF7289	4300	Ladera SW del Volcán Chiles	Carchi	EC
A-P01245	17NRA3310	4700	Ladera SW del Nevado Cayambe	Pichincha	EC
A-P01273	17NQA9539	4500	Ladera Sur del Nevado Cotacachi	Imbabura	EC
A-P01283	17MQV5527	4500	Ladera E del volcán Iliniza	Pichincha	EC
A-P02243	17MQU8438	4300	Volcán Tungurahua	Tungurahua	EC

Sup-4: Upper dry Ecuadorian super-páramo

Code VegPáramo	*UTM coord. (1Km)*	*Elevation (m)*	*Locality*	*Province*	*Country*
A-P01300	17MRV1545	4600	Ladera W del Antisana	Napo	EC
A-P01313	17MQV8427	4500	Ladera N del Cotopaxi	Pichincha	EC
A-P02599	17MQU3835	4605	ladera Norte del Volcan Chimborazo	Chimborazo	EC
A-P02601	17MQU3241	4257	El Arenal	Bolívar	EC
A-P02614	17MQU3833	4444	ladera SW del Chimborazo	Chimborazo	EC
