# Supplementary figures and images for: Phytoregionalisation of the Andean páramo
Source: PeerJ. 2018 Jun 1;6:e4786. doi: 10.7717/peerj.4786 (PMC5985761; doi:10.7717/peerj.4786)

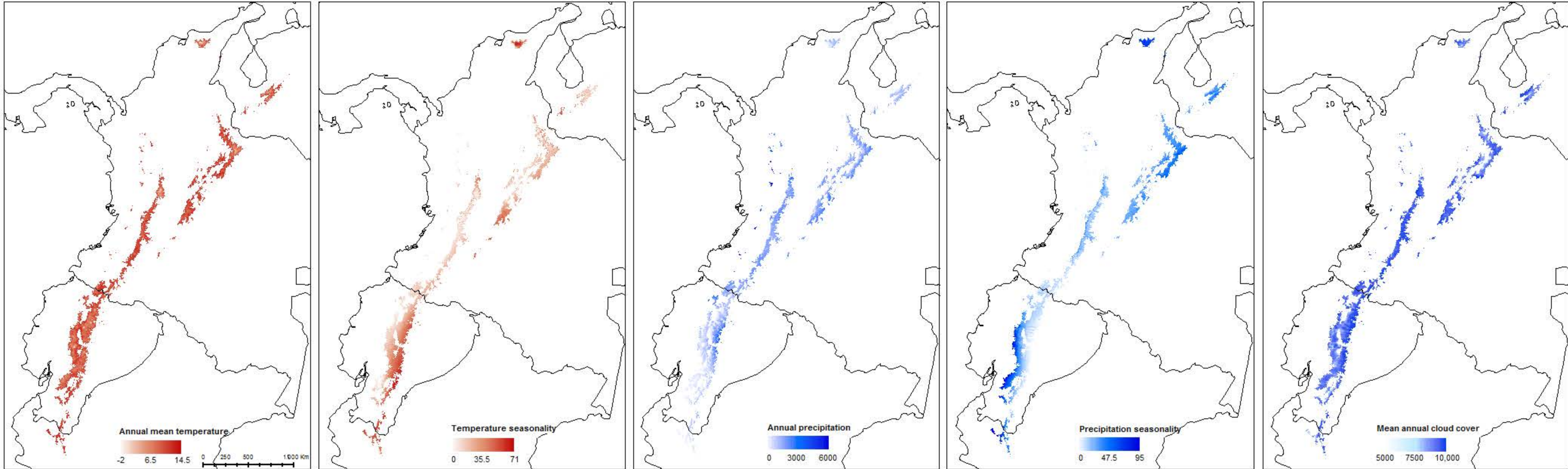

Supplemental information SI. 2 to Peyre et al. Phytoregionalisation of the Andean páramo

Supplement: Supplemental Information 2 — From left to right: annual temperature, temperature seasonality, annual precipitation, precipitation seasonality, cloud cover. All variables were obtained from the CHELSA 1.2 project. [file peerj-06-4786-s002.pdf]
