# Supplementary material for: Phytoregionalisation of the Andean páramo
Source: PeerJ. 2018 Jun 1;6:e4786. doi: 10.7717/peerj.4786 (PMC5985761; doi:10.7717/peerj.4786)
Supplement: Supplemental Information 3 [file peerj-06-4786-s003.pdf]

Supplemental information SI. 3 to Peyre et al. Phytoregionalisation of the Andean páramo

Cluster	VegPáramo plot	Latitude	Longitude	Elevation	Locality	Province/Department	Country
Mid1	A.P00361	6,4718766	-76,089749	3500	Páramo de Frontino	Antioquía	Colombia
Mid1	A.P00463	1,2695569	-77,8348304	3470	Volcán Cumbal	Nariño	Colombia
Mid1	A.P00599	5,0971077	-76,0690706	3490	Páramo de Tatamá	Risaralda	Colombia
Mid1	A.P00778	6,0983581	-72,9715197	4220	Páramo de Guantiva	Boyacá	Colombia
Mid1	A.P00875	5,9721771	-73,0803491	3725	Páramo La Rusia	Boyacá	Colombia
Mid1	A.P01546	4,5723086	-73,8055798	3350	Parque Nacional Natural de Chingaza	Cundinamarca	Colombia
Mid1	A.P02062	4,7993092	-75,455418	4400	Nevado de Santa Rosa	Risaralda	Colombia
Mid2	A.P00877	6,0620252	-72,9264994	3605	Páramo de Guantiva	Boyacá	Colombia
Mid2	A.P01572	5,5916304	-72,8470102	3400	Páramo de la Sarna	Boyacá	Colombia
Mid2	A.P02723	8,0272293	-71,970799	3273	Páramo las Rosas	Táchira	Venezuela
Mid2	A.P02787	7,9554126	-72,0841539	3120	Páramo el Zumbador	Tachira	Venezuela
Mid3	A.P00868	5,9812498	-73,0893482	3800	Páramo de la Rusia	Boyacá	Colombia
Mid3	A.P00879	5,55928308	-72,69863956	3605	Páramo de Tota	Boyacá	Colombia
Mid3	A.P00883	4,567875413	-74,05369948	4110	Sierra Nevada del Cocuy	Boyacá	Colombia
Mid3	A.P01901	4,2833978	-74,2115066	3440	Macizo de Sumapaz	Cundinamarca	Colombia
Mid3	A.P01919	3,9395696	-74,1307799	3540	Macizo de Sumapaz	Meta	Colombia
Mid4	A.P00432	1,079906	-77,7179298	3960	Volcán Azufral	Nariño	Colombia
Mid4	A.P00441	0,835758	-77,9242017	3800	Volcán Chiles	Nariño	Colombia
Mid4	A.P00853	6,0173516	-73,0711605	3795	Páramo de la Rusia	Boyacá	Colombia
Mid4	A.P00858	5,9791032	-72,5476129	3620	Páramo de Pisva	Boyacá	Colombia
Mid4	A.P00870	6,098117	-72,9082983	3765	Páramo de Guantiva	Boyacá	Colombia
Mid4	A.P01414	2,8990279	-76,1471856	3700	Páramo de Santo Domingo	Cauca	Colombia
Mid4	A.P01511	4,4998446	-73,7426125	3650	Parque Nacional Natural de Chingaza	Cundinamarca	Colombia
Mid4	A.P01918	4,2833104	-74,1304098	3750	Macizo de Sumapaz	Cundinamarca	Colombia
Mid4	A.P02575	-0,347923	-78,2016842	3945	Páramo de Papallacta	Pichincha	Ecuador
Mid4	A.P02758	9,0207505	-70,5238626	3383	Páramo de Guirigay	Trujillo	Venezuela
Mid5	A.P00471	1,2695371	-77,8527859	3600	Volcán Cumbal	Nariño	Colombia
Mid6	A.P00422	1,2158082	-77,3498839	4050	Volcán Galeras	Nariño	Colombia
Mid6	A.P02578	0,1310185	-78,2735403	3774	Laguna de Mojanda	Imbabura	Ecuador
Mid6	A.P02633	-0,6554095	-78,6774031	3787	Cerro Iliniza	Cotopaxi	Ecuador
Mid6	A.P02645	-0,5197949	-78,6325616	3742	Cerro Corazón	Pichincha	Ecuador
Mid9	A.P02327	-0,3027429	-78,2106729	4000	Cerro Guamaní	Pichincha	Ecuador
Mid9	A.P02353	0,3659949	-78,3183774	3600	Volcán Cotacachi	Imbabura	Ecuador
Sub2	A.P00465	1,070869	-77,7179218	3520	Volcán Azufral	Nariño	Colombia
Sub2	A.P00673	10,2565651	-72,941308	3077	Montaña de Perijá	César	Colombia
Sub2	A.P00864	5,2148258	-74,0029328	3675	Páramos de Neusa	Cundinamarca	Colombia
Sub2	A.P00869	5,9812498	-73,0893482	3800	Páramo de la Rusia	Boyacá	Colombia
Sub2	A.P00881	2,848931524	-76,12471282	3780	Páramo de Sumapaz	Meta	Colombia

Cluster	VegPáramo plot	Latitude	Longitude	Elevation	Locality	Province/Department	Country
Sub2	A.P01045	8,815256136	-70,86418281	4400	Páramo de Piedras Blancas	Mérida	Venezuela
Sub2	A.P01261	-0,4834321	-78,1118763	4600	Volcán Antisana	Napo	Ecuador
Sub2	A.P01936	3,9305143	-74,1217825	3650	Nevado de Sumapaz	Cundinamarca	Colombia
Sub2	A.P02577	0,1310176	-78,2645637	3797	Laguna de Mojanda	Imbabura	Ecuador
Sub2	A.P02584	-1,4506604	-78,4432949	3888	Volcán Tungurahua	Tungurahua	Ecuador
Sub2	A.P02622	-2,7807109	-79,2233141	3962	Parque Nacional El Cajas	Azuay	Ecuador
Sub2	A.P02663	-1,496055	-78,6228722	4042	Cerro Igualita	Tungurahua	Ecuador
Sub2	A.P02667	-1,6409324	-78,8383093	3888	Vía San Juan-Vinchoa	Chimborazo	Ecuador
Sub2	A.P02677	-2,30985	-78,7745317	3728	Páramo parroquia Achupallas	Chimborazo	Ecuador
Sub2	A.P02679	-3,7122645	-79,3027001	3467	Páramo carretera Loja-Saraguro	Loja	Ecuador
Sub2	A.P02694	-0,8725041	-78,910795	3753	Laguna Quilotoa	Cotopaxi	Ecuador
Sub2	A.P02718	8,018196	-71,9707333	3225	Páramo las Rosas	Táchira	Venezuela
Sup1	A.P00837	3,9395506	-74,1127663	4130	Páramo de Sumapaz	Meta	Colombia
Sup2	A.P00013	6,538351	-72,3281386	4100	Sierra Nevada del Cocuy	Boyacá	Colombia
Sup2	A.P00416	1,2158082	-77,3498839	4250	Cerro Galeras	Nariño	Colombia
Sup2	A.P00900	4,477039663	-73,78344923	4280	Sierra Nevada del Cocuy	Boyacá	Colombia
Sup2	A.P02415	-1,2608753	-78,4614545	4300	Volcán El Altar	Chimborazo	Ecuador
Sup3	A.P00901	6,3666144	-72,3200042	4310	Sierra Nevada del Cocuy	Boyaca	Colombia
Sup3	A.P02312	-3,160163	-79,0338005	3200	Estación de Cumbe	Azuay	Ecuador
Sup4	A.P01298	-0,6554263	-78,7133233	4600	Cerro Antisana	Napo	Ecuador
