# Supplementary material for: Phytoregionalisation of the Andean páramo
Source: PeerJ. 2018 Jun 1;6:e4786. doi: 10.7717/peerj.4786 (PMC5985761; doi:10.7717/peerj.4786)
Supplement: Supplemental Information 4 — Percentage of presence (> 1%) of each species within each cluster, corresponding to a phytogeographical unit. Sub-1: Guaramacal sub-páramo, Sub-2: Widespread sub-páramo, Mid-1: Périja-Santa Marta mid-páramo, Mid-2: Eastern cordillera mid-páramo, Mid-3: Central and western cordilleras mid-páramo, Mid-4: Mixed group of humid mid-páramo , Mid-5: Carchi mid-páramo, Mid-6: Ecuadorian mid-páramo, Mid-7: Venezuelan mid-páramo and lower super-páramo, Mid-8: The Nevados upper mid-páramo, Mid-9: The Ecuadorianuppermid-páramo, Sup-1: Lower humid super-páramo, Sup-2: The Nevados super-páramo, Sup-3: Upper humid super-páramo, Sup-4: Upper dry Ecuadorian super-páramo.. [file peerj-06-4786-s004.pdf]

Supplemental information SI. 4 to Peyre et al. Phytoregionalisation of the Andean páramo

Taxon Name	Sub-1	Sub-2	Mid-1	Mid-2	Mid-3	Mid-4	Mid-5	Mid-6	Mid-7	Mid-8	Mid-9	Sup-1	Sup-2	Sup-3	Sup-4
Pernettya prostrata (Cav.) DC.	81	22	48	45	37	85	70	82	33	55	44	58	8	22	1
Hypochaeris sessiliflora Kunth		3	32	16		6	30	56		58	75	67	74	48	65
Calamagrostis effusa (Kunth) Steud.	2	1	78	91	73	69	100	3	8	47		3	1		
Luzula racemosa Desv.		1	9		4	4		3	41	19	40	38	48	62	18
Calamagrostis intermedia (J.Presl) Steud.		17	16				5	88			65	43		5	39
Lycopodium clavatum L.	92	16	10	43	11	11	50	23	8		2				
Geranium sibbaldioides Benth.		8	13	11	7	42	12	47		48	5	51	8	3	
Oritrophium peruvianum (Lam.) Cuatrec.		4	3	15	12	11	9	41		47	2	57	9	35	4
Disterigma empetrifolium (Kunth) Drude		7		7	31	14	50	50		11	6	49	4	19	
Bromus lanatus Kunth		1			1			17	1	41	50	28	58	7	32
Baccharis caespitosa (Ruiz & Pav.) Pers.								10		16	51	19	25	55	60
Castilleja fissifolia L.f.	5	8	30	18	7	6	16	11	41	34	17	14	4	14	4
Agrostis tolucensis Kunth		3	22	2				21	31	19	26	10	1	11	68
Xenophyllum humile (Kunth) V.A. Funk					3	2		12		8	7	89	29	54	1
Eryngium humile Cav.		9	2	2	9	7	7	43	10	48	35	19	2	9	1
Hypericum laricifolium Juss.		12	3	1	43	7	54	5	43	22	7				
Oreomyrrhis andicola (Kunth) Hook. f.		2			1			15		44	49	44	23	6	8
Valeriana microphylla Kunth		9			4		3	52			39	52		17	13
Gentiana sedifolia Kunth			1	2	4	4		25		26	44	45	13	17	
Huperzia crassa (Humb. & Bonpl. ex Willd.) Rothm.			1		3		7	4		13	10	54	40	45	3
Blechnum loxense (Kunth) Hook. ex Salomon		5	2	36	50	26	40	7	1		5			5	
Agrostis foliata Hook.f.		1						5	1	4	5	47	33	75	6
Rhynchospora macrochaeta Steud. ex Boeck.	42	12	6	57	20	26		7			2				
Nertera granadensis (Mutis ex L.f.) Druce	13	10	9	8	27	25	25	11	1	5	7	8		3	
Cerastium floccosum Benth.								1		6	5	9	40	48	42
Arcytophyllum nitidum (Kunth) Schldl.	28	8	31	60	3	4			10			5			
Poa cucullata Hack.								15			29	36		52	16
Valeriana pilosa Ruiz & Pav.		5	10	2	6	9				30	1	29	45	8	
Halenia weddelliana Gilg		3			2		40	34			36	27		1	
Carex pichinchensis Kunth			5	5	12	30	29	16		22	6	16			
Azorella aretioides (Kunth) Willd. ex DC.					3		5	17		5	19	65		23	4
Pentacalia vaccinioides (Kunth) Cuatrec.			10	5	53	18	45	1		8					
Hypericum juniperinum Kunth	28	3	15	45	13	30			5						
Vaccinium floribundum Kunth		18	10	32	21	9	23	9	6	5	3		2		
Bidens triplinervia Kunth		8	15			2	7	28	20	1	33				21
Paspalum bonplandianum Flüggé		9	10	10		4	25	58	4		13			1	
Chusquea tessellata Munro	5	3	10	42	35	34				1			1		
Lachemilla orbiculata (Ruiz & Pav.) Rydb.		7			4	3		25		19	59	8	1		
Oreobolus goeppingeri Suess.		1		14			76	30				4			
Azorella pedunculata (Spreng.) Mathias & Constance		2						13			72	3		34	
Hieracium avilae Kunth	7	3	26	10	15	6	7		9	37			3		
Galium hypocarpium (L.) Endl. ex Griseb.	2	18	12	5	6	6	3	6	43	11	10				1
Lachemilla hispidula (L. M. Perry) Rothm.		1	5		3	6		4		1	3	58	1	39	
Jamesonia imbricata (Sw.) Hook. & Grey.	34	1	1	19	9	8	43		2						
Gnaphalium antennarioides DC.		1	21		1	1	3		6	61	3	1	17		
Melpomene moniliformis (Lag. ex Sw.) A.R. Sm. & R.C. Moran	7	8	6	8	7	8			5	6	1	6	16	35	
Carex pygmaea Boeck.		1	20	10	6	13	3	32		5	17	2	3		
Arcytophyllum muticum (Wedd.) Standl.			5	26	4	65		6	1	2			2		
Lupinus microphyllus Desr.					1		1	2		37	31	6	7	6	19
Rumex acetosella L.		1	5		3	4		5	42	26	19		4		
Sisyrinchium jamesonii Baker				12	13	4	23	28	8		6	12		2	
Espeletia pycnophylla Cuatrec.					1		100	6				1			
Lasiocephalus ovatus Schltdl.							5	3			21	21		34	24
Cortaderia hapalotricha (Pilg.) Conert	89	1							16						
Calamagrostis bogotensis (Pilg.) Pilg.		4	3	51	6	14	7	8	8		1	1		1	
Lachemilla nivalis (Kunth) Rothm.		2	1	2	2	11	7	7		15		25	25	7	
Senecio nivalis (Kunth) Cuatrec.											3	3		69	29
Erigeron ecuadoriensis Hieron.											24	27		51	1
Senecio formosus Kunth		2		9	3	7			41	37			3		
Chuquiraga jussieui J.F. Gmel.		2						1			29	4			63
Calamagrostis recta (Kunth) Trin. ex Steud.			5							72	1	3	16		
Senecio canescens (Bonpl.) Cuatrec.					1			1			3	3	37	34	18
Hesperomeles obtusifolia (Pers.) Lindl.	25	24	2	2	9	2	1		27		4				
Clinopodium nubigenum (Kunth) Kuntze			3		2			18	1	38	15	11		5	1
Plantago rigida Kunth						1				18	17	52	3	3	
Acaena cylindristachya Ruiz & Pav.		2	45	1		3			42						
Orthrosanthus chimboracensis (Kunth) Baker		13	29	1	1	6	7	21	11		3				

Espeletia hartwegiana Sch. Bip.					29	1				56			3		
Hypericum lancioides Cuatrec.				2	7	8	9	1	4	34	2	10	11		
Loricaria thuyoides (Lam.) Sch. Bip.		1			5		29	2			9	29		8	3
Werneria nubigena Kunth		1						7			50	1		1	26
Baccharis tricuneata (L.f.) Pers.		3	5	9	4	24		2	4	33	1				
Niphogeton dissecta (Benth.) J.F.Macbr.		4	9		5	1		6	5	12	7	30	4		
Festuca dolichophylla J. Presl		2	7	16	4	19				25	4		6		
Rhynchospora ruiziana Boeck.		5			9		45	19	4						
Espeletia grandiflora Humb. & Bonpl.				56		21				1					
Bartsia pedicularoides Benth.									3	43	2	6	23		
Chusquea angustifolia (Soderstr. & C.E.Calderón) L.G.Clark	73	3													
Erigeron chionophilus Wedd.										27			49		
Pentacalia vernicosa (Sch. Bip. ex Wedd.) Cuatrec.					10	8				44			12		
Puya hamata L.B. Sm.							63	10			1				
Paepalanthus karstenii Ruhland	2	1	7	32	2	25			3				1		
Pentacalia andicola (Turcz.) Cuatrec.		2	2	1	3		34	8	5	1	4	12			
Elaphoglossum mathewsii (Fée) T. Moore		2	1		9			2	3	8	10	6	7	21	1
Diplostephium rupestre (Kunth) Wedd.					9	1		1		4	2	40	6	7	
Disterigma acuminatum (Kunth) Nied.	52	10			6				1						
Belloa kunthiana (DC.) Anderb. & S.E.Freire		1		2					5	29	3	12	15	2	
Uncinia macrolepis Decne.		1			1			7		12	26	21		1	
Azorella corymbosa (Ruiz & Pav.) Pers.								1		1	2	35	1	28	1
Festuca ulochaeta Steud.		3						3		5	19	3	35		
Carex bonplandii Kunth	10	6	1	4	22	18			3	2					
Jamesonia goudotii (Hieron.) C. Chr.		1	1		6	1	3	5		12		24	7	6	
Werneria pumila Kunth		1								1	2	2		15	45
Eudema nubigena Humb. & Bonpl.												4		26	34
Polystichum orbiculatum (Desv.) J. Remy & Fée		1	1				1		6	6	7	6	2	27	6
Baccharis genistelloides (Lam.) Pers.		13			4			15		9	6			1	14
Ruilopezia lopez-palacii (Ruiz-Terán & López-Fig.) Cuatrec.	60														
Bartsia santolinifolia (Kunth) Benth.			3	35	2	12			1	6			1		
Diplostephium phylicoides (Kunth) Wedd.			2	57		1									
Senecio rhizocephalus Turcz.								5		29	4	8	14		
Agrostis breviculmis Hitchc.		1			1			1	2	9	34	8	3		
Cortaderia nitida (Kunth) Pilg.		3		3	22	5	9	2	3		9	1		1	
Poa pauciflora Roem. & Schult.		1				1		21	9	9	9	5	2		1
Xyris subulata Ruiz & Pav.	47		1	5		1		3							
Chaetolepis lindeniana (Naudin) Triana	31	3	3						20						
Blechnum schomburgkii (Klotzsch) C. Chr.	44						12								
Ranunculus praemorsus Kunth ex DC.		2						5	1	12	26	7	2	1	
Espeletia schultzii Wedd.		1							55						
Diplostephium schultzii Wedd.					28					25			3		
Lachemilla galioides (Benth.) Rothm.					7	1		4		40	1	3			
Diplostephium rhododendroides Hieron.							56								
Bartsia laticrenata Benth.							1	18			16	17		4	
Calamagrostis fibrovaginata Laegaard								6	9		25	11	1	4	
Galium corymbosum Ruiz & Pav.		6			1			22			20				6
Astragalus geminiflorus Humb. & Bonpl.											1				54
Geranium diffusum Kunth		2						7	24		21				
Luzula gigantea Desv.		7	3		4	6			2	11	5	8	2	4	
Cerastium subspicatum Wedd.					2					27			23		
Ctenopteris flabelliformis (Poir.) J. Sm.	2	8							3			17		21	
Sibthorpia repens (L.) Kuntze		10	1		6	4		16		1	10	3			
Geranium stramineum Triana & Planch.		2			13			1		4	4	25		1	1
Aetheolaena lingulata (Schltdl.) B. Nord.								8			10	13		2	18
Carex peucophila Holm				5		1				37			7		
Lachemilla holosericea (L.M. Perry) Rothm.					1					22	3	10	12	2	
Hypericum caracasanum Willd.	28	1	5			1			14						
Sisyrinchium tinctorium Kunth		3		2		1		9	33	1					
Gentianella corymbosa (Kunth) Weaver & Rödenberg			8	24	10	4			3						
Valeriana alypifolia Kunth												1		5	42
Monticalia peruviana (Pers.) C. Jeffrey		3						11			25	7		1	
Calamagrostis planifolia (Kunth) Trin. ex Steud.		1		16		1			28		1				
Cotula mexicana (DC.) Cabrera		1			6	4				19	10	7			
Castilleja nubigena Kunth											5	10		5	26
Conyza popayanensis (Hieron.) Pruski		1	9						8	27					
Calamagrostis ligulata (Kunth) Hitchc.		1								1		3	9	31	
Polypogon exasperatus (Trin.) Renvoize			9	2		4			5	16	1	1	7		
Lupinus pubescens Benth.							45								
Hypericum paramitanum N.Robson	44														
Hydrocotyle bonplandii A.Rich.		6			3	4	1	9	2		19				

Plantago sericea Ruiz & Pav.		2	5						3		25				9
Hieracium jubatum Fr.		6					16	2	9		10				
Brachyotum lindenii Cogn.					5		38								
Calamagrostis mollis Pilg.											9				34
Hypericum decandrum Turcz.		6						36							
Ophioglossum crotalophoroides Walter					1	3		2		8	3	9	1	15	
Lycopodium magellanicum (P. Beauv.) Sw.		1				1		26	2		1			10	
Castratella piloselloides (Bonpl.) Naudin			3	35		3									
Escallonia myrtilloides L.f.		8			17	4				11					
Olsynium trinerve (Baker) R.A. Rodr. & Martic.		1	1		1	1	9			26			1		
Ranunculus peruvianus Pers.					4	2	3	19		4	5	3			
Ourisia muscosa Benth.										2	2	5	4	27	
Geranium stoloniferum Standl.	39														
Oreobolus venezuelensis Steyerm.	15			14	3	6			1						
Agrostis magellanica Lam.		1						1		2		5	30		
Agrostis araucana Phil.			5	2	1						1		30		
Puya santosii Cuatrec.				26	3	10									
Stevia lucida Lag.		3	14						21						
Cortaderia sericantha (Steud.) Hitchc.		1	2	4	6		23			1		1			
Azorella crenata (Ruiz & Pav.) Pers.			5	2	1	1		5		23		1			
Geranium multipartitum Benth.											34				4
Gaultheria anastomosans (Mutis ex L.f.) Kunth	13	8		12	3	1									
Gaultheria erecta Vent.	2	13	11	5	3			2	1						
Geranium reptans R. Knuth		6						7			18	3		2	1
Aragoa abietina Kunth				29		8									
Lachemilla mandoniana (Wedd.) Rothm.					1	5		2	2	4	7	8	1	7	
Diplostephium eriophorum Wedd.					1	3				15			18		
Geranium humboldtii Spreng.								16			16	5			
Plantago linearis Kunth								14		1	19				1
Calandrinia acaulis Kunth										20	5		7	3	
Myrsine dependens (Ruiz & Pav.) A.Spreng.	7	11	1		4	1		2	8						
Lobelia tenera Kunth		5	3	4		1		2	18		1				
Festuca procera Kunth			1		4					19	3		7		
Halenia campanulata Cuatrec.					12					5			17		
Nototriche jamesonii A.W. Hill															34
Gentianella nummularifolia (Griseb.) Fabris								4			2	21		6	
Gentianella dasyantha (Gilg.) Fabris										25			8		
Silene thysanodes Fenzl											13			1	19
Loricaria complanata (Sch. Bip.) Wedd.		1	1		11	1		2				12	3	1	
Lachemilla uniflora Maguire			1					27			1	3			
Gnaphalium pensylvanicum Willd.								23			8			1	
Rhynchospora guaramacalensis M.T.Strong	31														
Daucus montanus Humb. & Bonpl. ex Schult.	23	1							2		5				
Gaiadendron punctatum (Ruiz & Pav.) G.Don		6	4	2	9		10								
Lachemilla vulcanica (Schltdl. & Cham.) Rydb.		2									26	3			
Gaylussacia buxifolia Kunth		1	14	15		1									
Baccharis prunifolia Kunth		1	1		1	1			27						
Halenia asclepiadea (Kunth) G. Don		1		20	1	8						1			
Phyllactis rigida (Ruiz & Pav.) Pers.			2					8			21				
Viola bangii Rusby								4			8	5		1	13
Gentianella cerastioides (Kunth) Fabris											31				
Orthrosanthus acorifolius (Kunth) Ravenna	10	1	2						17						
Acaena elongata L.		3	2		1	2			20		1		1		
Lachemilla sprucei (L.M.Perry) Rothm.		1	1						27		1				
Festuca asplundii E.B. Alexeev		1						2			2	25			
Hieracium sodiroanum Zahn								28			1			1	
Arenaria dicranoides Kunth												1		15	14
Oxalis spiralis Ruiz & Pav. ex G.Don		8							21						
Gunnera magellanica Lam.		4			3						7	15			
Gnaphalium americanum Mill.		2		1	1						15	8		2	
Geranium santanderiense R. Knuth				28		1									
Hypochaeris echegarayi Hieron.									29						
Poa subspicata (J.Presl) Kunth											11	7		3	8
Draba depressa Hook. f.														2	27
Jamesonia bogotensis H. Karst.		3	1	18	3	3									
Diplostephium floribundum (Benth.) Wedd.		1			10	1	16								
Muehlenbeckia volcanica (Benth.) Endl.		1							2	6	9		2	8	
Gnaphalium dombeyanum DC.			6					1	17		4				
Hinterhubera imbricata Cuatrec. & Aristeg.			1						27						
Lupinus prostratus J. Agardh								28							
Perezia pungens Less.								1			4			1	22

Achyrocline satureioides (Lam.) DC.		4	13					1	3		6				
Myrteola nummularia (Poir.) O.Berg				2	9		16								
Arenaria digyna Willd. ex Schltdl.						2				12			13		
Festuca glumosa Hack. ex E.B. Alexeev											4	10		13	
Echeveria bicolor (Kunth) Walth.		2	1						23						
Lupinus alopecuroides Desv.		2								1		1	20	2	
Agrostis trichodes (Kunth) Roem. & Schult.		1	8	2		2			13						
Azorella multifida (Ruiz & Pav.) Pers.		1			1	1				12	9		2		
Chrysactinium acaule (Kunth) Wedd.		1						20			4			1	
Senecio chionogeton Wedd.		1						18			4	3			
Gynoxys buxifolia (Kunth) Cass.		5					16				4				
Lachemilla andina (L.M. Perry) Rothm.		5					7	6	2		5				
Geranium multiceps Turcz.				10	1	1			13						
Senecio comosus Sch. Bip.										4		3	6	12	
Senecio latiflorus Wedd.										4			20	1	
Pentacalia gelida (Wedd.) Cuatrec.										1			24		
Draba aretioides Humb. & Bonpl. ex DC.											3			22	
Paepalanthus alpinus Körn		3		20		1									
Baccharis macrantha Kunth		1	2		15	1	1			1			1	2	
Diplostephium hartwegii Hieron.					10		1	13							
Senecio isabelis S. Díaz					1					6			17		
Pinguicula calyptrata Kunth							14	10							
Poa petrosa Swallen									24						
Cerastium imbricatum Kunth										2	21			1	
Festuca andicola Kunth											23	1			
Draba obovata Benth.											9			7	8
Conyza cardaminifolia Kunth											6				18
Loricaria ilinissae (Benth.) Cuatrec.											3	10			11
Vaccinium corymbodendron Dunal	18				1				4						
Arcytophyllum setosum (Ruiz & Pav.) Schltdl.		7					3	2			1	10			
Dorobaea pimpinellifolia (Kunth) B.Nord.		5					1	11			4			2	
Gnaphalium purpureum L.		4							17		1	1			
Bartsia crisafullii N.H. Holmgren		3						20							
Lachemilla aphanoides (Mutis ex L.f.) Rothm.		2	5	1	9				5	1					
Bartsia orthocarpiflora Benth.		1			14			1			2	5			
Monticalia arbutifolia (Kunth) C. Jeffrey		1			7						10	5			
Carex toreadora Steyerm.											9	14			
Nototriche phyllanthos (Cav.) A.W. Hill											6			6	11
Anthoxanthum odoratum L.		3					1	2		2	13		1		
Valeriana bracteata Benth.		1			1			2				17		1	
Aphanactis villosa S.F. Blake		1						16			3	1			1
Colobanthus quitensis (Kunth) Bartl.										8	3		4	7	
Carex aciculares (Kük.) G.A. Wheeler												21		1	
Aciachne flagellifera Laegaard												20	2		
Ugni myricoides (Kunth) O.Berg	10			4	7										
Carex jamesonii Boott	2	2		2	9	2				2	2				
Stellaria recurvata Willd. ex Schltdl.		6						2			12	1			
Equisetum bogotense Kunth		3			8			5	1		4				
Brachypodium mexicanum (Roem. & Schult.) Link		2							16		3				
Hypericum goyanesii Cuatrec.		1		8		12									
Aciachne acicularis Laegaard									21						
Gentianella cernua (Kunth) Fabris											2	8			11
Gaultheria hapalotricha A.C.Sm.	15			5											
Cortaderia columbiana (Pilg.) Pilg.		1	5	9					5						
Lachemilla tanacetifolia Rothm.		1						2		2		1	7	7	
Arenaria venezuelana Briq.		1							19						
Oreobolus obtusangulus Gaudich.			1	10	3	4		2							
Plantago major L.								10			3	7			
Aphanactis jamesoniana Wedd.										12	7		1		
Festuca vaginalis (Benth.) Lægaard											2				18
Elaphoglossum lingua (C. Presl) Brack.	13	6													
Festuca tolucensis Kunth		3							16						
Cestrum buxifolium Kunth		2	3						14						
Lourteigia stoechadifolia (L.f.) R.M.King & H. Rob.		1	15						3						
Distichia muscoides Nees & Meyen		1			3		1					12	2		
Coespeletia moritziana (Sch.Bip.) Cuatrec.		1							18						
Noticastrum marginatum (Kunth) Cuatrec.			5			3			11						
Pseudognaphalium moritzianum (Klatt) V.M.Badillo									19						
Montia fontana L.											3	2		14	
Viola polycephala H.E. Ballard & P. Jørg.															19
Chusquea steyermarkii L.G.Clark	18														

Libanothamnus griffinii (Ruiz & López) Cuatrec.	18														
Pteridium arachnoideum (Kaulf.) Maxon		15	3												
Macleania rupestris (Kunth) A.C.Sm.		12		4	2										
Weinmannia cochensis Hieron.		10			1		7								
Cerastium danguyi J.F. Macbr.		1						11			4	2			
Pentacalia microdon (Wedd.) Cuatrec.		1									1			2	14
Loricaria colombiana Cuatrec.					6					1			11		
Oritrophium hieracioides (Wedd.) Cuatrec.								7			4	7			
Geranium maniculatum H.E. Moore								4				13		1	
Trisetum spicatum (L.) K.Richt.								3	1		7			3	4
Lasiocephalus longipenicillatus (Sch.Bip. ex Sandwith) Cuatrec.									18						
Oxylobus glanduliferus (Sch.Bip. ex Benth. & Hook.f.) A.Gray									18						
Belloa radians (Benth.) Sagàst. & Dillon									11		1				6
Aretiastrum aretioides (Kunth) Graebn.											1	13		3	1
Calamagrostis ecuadoriensis Lægaard												14		4	
Geranium ecuadoriense Hieron.															18
Puya aristeguietae L.B.Sm.	15								2						
Agrostis perennans (Walter) Tuck.		5				1		5	2		4				
Bomarea linifolia (Kunth) Baker		1			15						1				
Sisyrinchium pusillum Kunth			13	3							1				
Luciliocline longifolia (Cuatrec. & Aristeg.) M.O.Dillon & Sagást.			5						10		2				
Oritrophium limnophilum (Sch.Bip.) Cuatrec.								2	1		1	8		4	1
Baccharis rupicola Kunth										16			1		
Lupinus smithianus Kunth											3				14
Achyrocline alata (Kunth) DC.		6	5		1	1					3				
Hypericum aciculare Kunth		3						10				3			
Lachemilla hirta (L.M.Perry) Rothm.		2						1	9		4				
Diplostephium cinerascens Cuatrec.		1			9					5			1		
Hypericum magdalenicum N. Robson			16												
Pentacalia albotecta (Cuatrec.) Cuatrec.			16												
Espeletia argentea Humb. & Bonpl.			5	5		6									
Espeletia frontinoensis Cuatrec.					4	12									
Chaptalia cordata Hieron.							16								
Chusquea aristata Munro							14	1				1			
Calamagrostis guamanensis Escalona								1				13		2	
Draba hallii Hook. f.										4	2	1	8	1	
Valeriana adscendens Turcz.												12		4	
Pentacalia cachacoensis (Cuatrec.) Cuatrec.	13	2													
Blechnum auratum (Fée) R.M. Tryon & Stolze	5	6		3	1										
Lycopodium jussiaei Desv. ex Poir.		6	4			1				4					
Puya nitida Mez		3		10				2							
Monnina crassifolia (Bonpl.) Kunth		2					7	2			4				
Stellaria cuspidata Willd. ex Schlecht.		1			5	1				2	6				
Draba pulvinata Turcz.		1							14						
Bejaria resinosa Mutis ex L.f.			7	8											
Lachemilla moritziana Damm.			4						11						
Niphogeton ternata (Willd. ex Schult.) Mathias & Constance					15										
Calamagrostis jamesonii Steud.											5	6		4	
Lachemilla rivulorum (Rothm.) Rothm.											5	4		6	
Loricaria antisanensis Cuatrec.												8		7	
Cybianthus marginatus (Benth.) Pipoly	10	4													
Disterigma alaternoides (Kunth) Nied.	10	2							2						
Peperomia galioides Kunth		9							5						
Baccharis buxifolia (Lam.) Pers.		4									10				
Arcytophyllum filiforme (Ruiz & Pav.) Standl.		3						9			1	1			
Calamagrostis pittieri Hack.		2							12						
Hypericum strictum Kunth		1	5						8						
Brachyotum strigosum (L. f.) Triana		1	2	7	3		1								
Halenia foliosa Gilg		1			2	11									
Lupinus purdieanus C.P. Sm.		1									4			9	
Ranunculus spaniophyllus Lourt.			14												
Carex confertospicata Boeck.			10	2		1							1		
Lachemilla polylepis (Wedd.) Rothm.			10						4						
Azorella julianii Mathias & Constance			2						12						
Calamagrostis macrophylla (Pilg.) Pilg.					14										
Myrosmodes paludosa (Rchb.f.) C.A.Vargas					5								9		
Lysipomia muscoides Hook. f.					1					6			7		
Hieracium adenocephalum (Sch. Bip.) Arv.-Touv.						14									
Halenia pulchella Gilg								2				9		3	
Ephedra rupestris Benth.											3				11
Lysipomia montioides Kunth											1	11		2	

Calamagrostis aurea (Munro ex Wedd.) Hack. ex Sodiro											2		8	4	
Gaultheria reticulata Kunth		11						2							
Aegopogon cenchroides Humb. & Bonpl. ex Willd.		5							8						
Hesperomeles ferruginea (Pers.) Benth.		4			4			1	4						
Rhynchospora aristata Boeck.		2	5		6										
Briza monandra (Hack.) Pilg.		2						8			3				
Cerastium mollissimum Poir.		2						2			9				
Espeletia perijaensis Cuatrec.			13												
Hypericum stenopetalum Turcz.			13												
Pentacalia abietina (Willd. ex Wedd.) Cuatrec.				9		4									
Lachemilla pectinata (Kunth) Rothm.					12						1				
Miconia pernettifolia Triana								13							
Viola dombeyana DC.								12			1				
Festuca chimborazensis E.B. Alexeev								3			6	4			
Carex amicta Boott									13						
Conyza filaginoides (DC.) Hieron.									13						
Gnaphalium meridanum Aristeg.									13						
Grammitis youngii Stolze												7		6	
Ageratina theaefolia (Benth.) R.M.King & H.Rob.	7	1	3						1						
Monnina salicifolia Ruiz & Pav.		4			8										
Ageratina gracilis (Kunth) R.M.King & H.Rob.		2	4	5		1									
Lachemilla jamesonii (L.M. Perry) Rothm.		1						2			9				
Jamesonia canescens Kunze			3			1			8						
Rhynchospora hieronymi Boeck.								12							
Cotula australis (Sieber ex Spreng.) Hook. f.								5			7				
Mona meridensis (Friedrich) Ö.Nilsson									4	5			3		
Plagiocheilus peduncularis (Kunth) Wedd.											7	5			
Lupinus rupestris Kunth														12	
Diplostephium obtusum S.F.Blake	10	1													
Ruilopezia jabonensis (Cuatrec.) Cuatrec.	10	1													
Muehlenbeckia tamnifolia (Kunth) Meisn.	2	7		1							1				
Hymenophyllum myriocarpum Hook.	2	7			2										
Gaultheria glomerata (Cav.) Sleumer	2	5	1				1		2						
Weinmannia fagaroides Kunth		11													
Clethra fimbriata Kunth		7		4											
Berberis goudotii Triana & Planch.		6		4		1									
Stachys elliptica Kunth		1				1		1			7				1
Geranium meridense Pittier		1							10						
Niphogeton glaucescens (Kunth) J.F. Macbr.			3	4	1	3									
Eleocharis stenocarpa Svenson					5	6									
Diplostephium revolutum S. F. Blake					3	8									
Calamagrostis coarctata Eaton					1				2				8		
Monticalia stuebelii (Hieron.) C. Jeffrey							5	5				1			
Jamesonia boliviensis A.F. Tryon								11							
Gentianella foliosa (Kunth) Fabris								2			4	5			
Poa trachyphylla Pilg.													11		
Rhynchospora gollmeri Boeckeler	10														
Ruilopezia paltonioides (Standl.) Cuatrec.	7	3													
Rubus acanthophyllos Focke	5			5											
Baccharis nitida (Ruiz & Pav.) Pers.		10													
Brachyotum naudinii Triana		10													
Lycopodium thyoides Humb. & BonpL ex Willd.		8			1				1						
Peperomia hartwegiana Miq.		7			2					1					
Elaphoglossum engelii (II. Karst.) H. Christ		6					1				3				
Triniochloa stipoides (Kunth) Hitchc.		4							6						
Vicia andicola Kunth		3				1		1	1		4				
Hypericum silenoides Juss.		3					7								
Cortaderia bifida Pilg.		2	6					2							
Carex lemanniana Boott		2						7				1			
Trifolium repens L.		2						1			7				
Hypericum mexicanum L.f.		1	6	2		1									
Diplostephium rosmarinifolium (Benth.) Wedd.		1	2	1	4	1							1		
Ourisia chamaedrifolia Benth.		1			1				2			5		1	
Halenia kalbreyeri Gilg		1						3			6				
Bejaria nana A.C.Sm. & Ewan			10												
Hypericum baccharoides Cuatrec.			10												
Lupinus carrikeri C. P. Sm.			10												
Pernettya hirta (Willd.) Sleumer				10											
Huperzia cruenta (Spring) Rothm.				5	3	2									
Monnina revoluta Kunth					10										
Miconia chionophila Naudin					4		5					1			

Arcytophyllum aristatum Standl.						2						7	1		
Lysipomia sphagnophila Griseb. ex Wedd.						1				4		3	2		
Xenophyllum crassum (S.F. Blake) V.A. Funk								3				1	1	4	1
Veronica serpyllifolia L.								2		8					
Bartsia laniflora Benth.									10						
Paspalum nutans Lam.									10						
Draba lindenii (Hook.) Planch. ex Sprague									2	5			3		
Aa colombiana Schltr.										9			1		
Myrrhidendron glaucescens (Benth.) J.M. Coult. & Rose										9			1		
Elaphoglossum yatesii (Sodiro) H. Christ														10	
Xenophyllum rigidum (Kunth) V.A. Funk														1	9
Chusquea spencei Ernst	2	7													
Calamagrostis tarmensis Pilg.		9													
Clinopodium obovatum (Ruiz & Pav.) Govaerts		9													
Paepalanthus meridensis Klotzsch ex Körn.		9													
Bomarea pauciflora (Kunth) Herb.		8		1											
Nassella inconspicua (J. Presl) Barkworth		4					3	1			1				
Vallea stipularis L.f.		4							5						
Paspalum trianae Pilg.		3				6									
Achyrocline lehmannii Hieron.		2	4	2					1						
Baccharis alaternoides Kunth		2						6			1				
Eccremis coarctata (Ruiz & Pav.) Baker		1	3			5									
Miconia salicifolia (Bonpl. ex Naudin) Naudin		1			5	2					1				
Terpsichore heteromorpha (Hook. & Grey.) A.R. Sm.		1			4							1		3	
Lachemilla verticillata (Field & Gardn.) Rothm.		1							8						
Oxalis tabaconasensis R.Knuth		1							8						
Trisetum irazuense (Kuntze) Hitchc.		1							8						
Oenothera multicaulis Ruiz & Pav.		1							3		5				
Elymus cordilleranus Davidse & R.W.Pohl		1									8				
Holodiscus argenteus (L.f.) Maxim.			9												
Lysipomia laciniata A.DC.			6	2									1		
Ageratina vacciniaefolia (Benth.) R.M.King & H.Rob.				6		3									
Oreobolus cleefii L. E. Mora				3	6										
Niphogeton lingula (Wedd.) Mathias & Constance				2		6				1					
Laestadia muscicola Sch.Bip. ex Wedd.						8				1					
Cerastium cephalanthum S.F.Blake									9						
Poa annua L.										6	2		1		
Draba pennell-hazenii O. E. Schulz										1			8		
Galium pumilio Standl.											9				
Jamesonia cinnamomea Kunze												9			
Saxifraga magellanica Poir.														6	3
Galium canescens Kunth	2	1				1			4						
Greigia collina L.B. Sm		8													
Morella pubescens (Humb. & Bonpl. ex Willd.) R.L.Wibur		7									1				
Ilex kunthiana Triana & Planch.		6			2										
Elaphoglossum minutum (Pohl ex Fée) T. Moore		6							2						
Chaetolepis microphylla (Bonpl.) Miq.		5	1	2											
Gaultheria rigida Kunth		5		3											
Arenaria lanuginosa (Michx.) Rohrb.		5						2	1						
Hypericum sprucei N. Robson		4						2			1	1			
Oxalis lotoides Kunth		3		5											
Desfontainia spinosa Ruiz & Pav.		3			5										
Cystopteris fragilis (L.) Bernh.		3									3			1	1
Miconia latifolia (D.Don) Naudin		2			6										
Bomarea multiflora (L.f.) Mirb.		2					3	1			2				
Arcytophyllum vernicosum Standl.		2						6							
Castilleja ecuadorensis N.H. Holmgren		2						6							
Bartsia melampyroides (Kunth) Benth.		2									6				
Danthonia secundiflora J.Presl		1	3	1					3						
Ribes andicola Jancz.		1		2		1			2		2				
Ranunculus nubigenus Kunth ex DC.		1			6	1									
Erigeron bonariensis L.		1			2	4			1						
Ceratochloa pitensis (Kunth) Holub		1							1		6				
Rumex tolimensis Wedd.		1								4			3		
Libanothamnus occultus (S.F.Blake) Cuatrec.			6						2						
Azorella cuatrecasasii Mathias & Constance			5			3									
Ortachne erectifolia (Swallen) Clayton			4			1			3						
Pentacalia nitida (Kunth) Cuatrec.			2	2		4									
Huperzia brevifolia (Grey. & Hook.) Holub				3		3		2							
Werneria pygmaea Gillies ex Hook. ex Arn.					1				1		2	1			3
Puya antioquensis L. B. Sm. & Read						8									

Laphangium luteoalbum (L.) Tzvelev.								7			1				
Bomarea glaucescens (Kunth) Baker								5			2	1			
Acaulimalva purdiaei (A.Gray) Krapov.									8						
Elaphoglossum inaequalifolium (Jenman) C. Chr.									8						
Potentilla heterosepala Fritsch									4	4					
Lepidium abrotanifolium Turcz.											8				
Baccharis arbutifolia (Lam.) Vahl											2	1		5	
Carex tamana Steyerm.											1	7			
Xenophyllum sotarense (Hieron.) V.A. Funk												2		6	
Valeriana quiroana Xena	7														
Chusquea laegaardii (L.G.Clark) L.G.Clark		7													
Clusia elliptica Kunth		7													
Elleanthus aurantiacus (Lindl.) Rchb.f.		7													
Lomatia hirsuta (Lam.) Diels		7													
Paspalum pilgerianum Chase		7													
Rhynchospora kunthii Nees ex Kunth		7													
Drimys granadensis L. f.		6							1						
Ageratina pichinchensis (Kunth) R.M.King & H.Rob.		3							3		1				
Espeletiopsis corymbosa (Humb. &. Bonpl.) Cuatrec.		2		5											
Ageratina tinifolia (Kunth) R.M.King & H.Rob.		2			2	1			2						
Hesperomeles goudotiana (Decnc.) Killip		1	3	2	1										
Eryngium humboldtii Delar		1	2	2		2									
Lachemilla fulvescens (Perry) Rothm.		1		3		1		1			1				
Gynoxys tolimensis Cuatrec.		1			6										
Cardamine bonariensis Juss. ex Pers.		1			2						3	1			
Stipa rosea Hitchc.		1						1			1				4
Draba cheiranthoides Hook. f.			7												
Paspalum hirtum Kunth			2	2		3									
Bucquetia glutinosa (L. f.) DC.				7											
Epidendrum chioneum Lindl.				7											
Miconia ligustrina (Sm.) Triana				7											
Isolepis inundata R.Br.					6	1									
Diplostephium glandulosum Hieron.					3			1			2	1			
Isoetes andina Spruce ex Hook.					2	2						3			
Carex peruviana J.Presl & C.Presl								7							
Geranium campii H. E. Moore								7							
Gnaphalium coarctatum Willd.								5			2				
Calamagrostis heterophylla (Wedd.) Pilg.									6		1				
Aciachne pulvinata Benth.										6			1		
Draba hookeri Walp.											7				
Arenaria jamesoniana Rohrb.											6			1	
Erigeron turczaninowii Wedd.											3				4
Castilleja pumila (Benth.) Wedd.											2	2		3	
Epidendrum frutex Rchb.f.	5				1										
Bejaria aestuans Mutis ex L.	2	4													
Serpocaulon funckii (Mett.) A.R. Sm.	2	4													
Geranium velutinum Turcz.	2	2	1						1						
Gentianella nevadensis (Gilg) Weaver & Rödenberg	2								4						
Brachyotum campanulare (Bonpl.) Triana		6													
Clethra ovalifolia Turcz.		6													
Hedyosmum luteynii Todzia		6													
Hydrocotyle humboldtii A. Rich.		6													
Lepanthes pteropogon Rchb. f.		6													
Oreocallis grandiflora (Lam.) R. Br.		6													
Persea ferruginea Kunth		6													
Pilea smithii Killip		6													
Polypodium mindense Sodiro		6													
Valeriana laurifolia Kunth		5								1					
Weinmannia microphylla Kunth		4		2											
Campyloneurum angustifolium (Sw.) Fée		4									2				
Epilobium denticulatum Ruiz & Pav.		3								2	1				
Chusquea spectabilis L.G.Clark		2			4										
Jamesonia pulchra Hook. & Grev.		2						4							
Cinna poiformis (Kunth) Scribn. & Merr.		1	5												
Cyperus compactus Retz.		1			2	3									
Pedicularis incurva Benth.		1						4		1					
Galium pseudotriflorum Dempster & Ehrend.		1						2			3				
Azorella biloba (Schltdl.) Wedd.		1						2				3			
Halenia viridis (Griseb.) Gilg		1							5						
Diplostephium tenuifolium Cuatrec.			5		1										
Pinguicula elongata Benj.			4		1	1									

Bartsia glandulifera Molau			2						4						
Rhynchospora marisculus Lindl. & Nees			1	4		1									
Arenaria musciformis Triana & Planch.			1						5						
Puya goudotiana Mez				3		3									
Paepalanthus pilosus (Kunth) Kunth						1		5							
Holcus lanatus L.							5				1				
Brachyotum alpinum Cogn.							1	5							
Ranunculus geranioides Kunth							1	1			4				
Chusquea rigida (L.G.Clark) L.G.Clark								6							
Gentianella hyssopifolia (Kunth) Fabris								6							
Uncinia lacustris G.A. Wheeler								5				1			
Uncinia phleoides (Cav.) Mey.								2			4				
Coespeletia timotensis (Cuatrec.) Cuatrec.									6						
Espeletia marthae Cuatrec.									6						
Festuca fragilis (Luces) B.Briceño									6						
Hinterhubera laseguei Wedd.									6						
Oenothera epilobiifolia Kunth									6						
Trisetum foliosum Swallen									6						
Belloa piptolepis (Wedd.) Cabrera									3						3
Gamochaeta humilis Wedd.											6				
Cerastium candicans Wedd.											5	1			
Cerastium trianae Briq.											5	1			
Calceolaria rosmarinifolia Lam.											3			3	
Calceolaria ferruginea Cav.											3				3
Aa argyrolepis Rchb. f.											1			5	
Isoetes novo-granadensis H.P. Fuchs												6			
Elaphoglossum ovatum (Hook. & Grey.) T. Moore												5		1	
Lachemilla rupestris (Kunth) Rothm.												5		1	
Eriosorus flexuosus (Kunth) Copel.	2	2				1									
Aetanthus colombianus A.C.Sm.		5													
Ageratina fastigiata (Kunth) R.M.King & H.Rob.		5													
Arcytophyllum rivetii Danguy & Cherm.		5													
Blechnum lima Rosenst.		5													
Calceolaria rotundifolia Kunth		5													
Chrysactinium caulescens (Hieron.) H. Rob. & Brettell		5													
Coreopsis oblanceolata S. F. Blake		5													
Miconia bullata (Turcz.) Triana		5													
Schizachyrium sanguineum (Retz.) Alston		5													
Stevia macbridei B. L. Rob.		5													
Sticherus revolutus (Kunth) Ching		5													
Geranium ayavacense Willd. ex Kunth		4	1												
Miconia elaeoides Naudin		4	1												
Bomarea brachysepala Benth.		4						1							
Miconia jahnii Pittier		3			2										
Dicksonia sellowiana Hook.		3						2							
Lupinus ramosissimus Benth.		3						2							
Oxalis medicaginea Kunth		3									2				
Lourteigia humilis (Benth.) R. M. King & H. Rob.		2	1	2											
Symplocos theiformis (L. f.) Oken		2		3											
Greigia mulfordii L.B. Sm.		2		2		1									
Hypochaeris radicata L.		2		1							2				
Lupinus expetendus C.P. Sm.		2			3										
Brachyotum ledifolium (Desr.) Triana		2						2			1				
Baccharis latifolia (Ruiz & Pav.) Pers.		2							3						
Bulbostylis tenuifolia (Rudge) J.F.Macbr.		1	4												
Geranium holosericeum Willd. ex Spreng.		1	4												
Jamesonia rotundifolia Fée		1			2			2							
Cerastium arvense L.		1			1	3									
Hesperomeles pernettyoides Wedd.		1			1				2	1					
Hieracium peruanum E. M. Fries		1						4							
Chaetolepis perijensis Wurdack			5												
Erigeron raphaelis Cuatrec.			5												
Jamesonia cuatrecasasii A. F. Tryon			5												
Lachemilla purdiei (L.M. Perry) Rothm.			5												
Pterichis galeata Lindl.			5												
Senecio leucanthemoides Cuatrec.			4			1									
Stenorrhynchos vaginatum (Kunth) Spreng.			3	2											
Hypericum phellos Gleason			3	1	1										
Espeletiopsis colombiana (Cuatrec.) Cuatrec.			3			2									
Puya trianae Baker			1	4											
Castilleja integrifolia L. f.				5											

Rhynchospora macrostachya Torr. ex A. Gray				5											
Gynoxys sancti-antonii Cuatrec.					3		1	1							
Lupinus humifusus Sessé & Moc. ex G. Don					3							2			
Lupinus colombiensis C. P. Sm.					2							3			
Gynoxys parvifolia Cuatrec.					1		3	1							
Hypericum thuyoides Kunth						5									
Niphogeton chirripoi (Suess.) Mathias & Constance						5									
Espeletia lopezii Cuatrec.						4							1		
Paepalanthus muscosus Körn.							5								
Halenia minima C.K. Allen								5							
Halenia taruga-gasso Gilg								5							
Ottoa oenanthoides Kunth								5							
Puya nutans L.B. Sm.								5							
Uncinia ecuadorensis G.A. Wheeler & Goetgh.								5							
Altensteinia fimbriata Kunth								3				2			
Berroa gnaphalioides (Less.) Beauverd								2			3				
Muhlenbergia ligularis (Hack.) Hitchc.								2			3				
Thelypteris caucaensis (Hieron.) Alston								2			3				
Gentianella hirculus (Griseb.) Fabris								2				3			
Espeletia jajoensis Cuatrec.									5						
Gnaphalium meridanum Aristeg.									5						
Lachemilla ramosissima (Rothm.) Rothm.									5						
Lepechinia bullata (Kunth) 004942									5						
Lupinus meridanus C.P.Sm.									5						
Pentacalia apiculata (Sch.Bip. ex Wedd.) Cuatrec.									5						
Draba rositae Santana & Rangel										2			3		
Taraxacum officinale F.H. Wigg.											5				
Gynoxys acostae Cuatrec.											3	2			
Calceolaria gossypina Benth.											2			3	
Carex collumanthus (Steyerm.) L.E.Mora												4		1	
Sphyrospermum buxifolium Poepp. & Endl.	2	2													
Themistoclesia dependens (Benth.) A.C.Sm.	2	1			1										
Bomarea setacea (Ruiz & Pav.) Herb.		4													
Calceolaria nivalis Kunth		4													
Diplostephium ochraceum (Kunth) Nees		4													
Disterigma pentandrum S.F. Blake		4													
Fuchsia petiolaris Kunth		4													
Geonoma undata Klotzsch		4													
Miconia aspergillaris (Bonpl.) Naudin		4													
Neonelsonia acuminata (Benth.) J.M. Coult. & Rose ex Drude		4													
Paranephelius uniflorus Poepp. & Endi.		4													
Thibaudia grantii A. C. Sm.		4													
Vaccinium crenatum (G. Don) Sleumer		4													
Weinmannia elliptica Kunth		4													
Oxalis integra R. Knuth.		3			1										
Bomarea uncifolia Herb.		3						1							
Brachyotum andreanum Cogn.		3						1							
Brachyotum confertum (Bonpl.) Triana		3						1							
Monticalia pulchella (Kunth) C. Jeffrey		2		2											
Miconia puracencis Wurdack		2			2										
Arracacia xanthorrhiza Bancr.		2						2							
Brachyotum benthamianum Triana		2						2							
Lophosoria quadripinnata (J. F. Gmel.) C. Chr.		1			3										
Rubus nubigenus Kunth		1			3										
Weinmannia mariquitae Szyszyl.		1			3										
Calceolaria microbefaria Kraenzl.		1			1				2						
Sisyrinchium micranthum Cav.		1				3									
Gaultheria buxifolia Willd.		1							3						
Paspalum prostratum Scribn. & Merr.		1							3						
Plantago australis Lam.		1							1		2				
Gentianella rapunculoides (Willd. ex Schult.) J.S. Pringle		1									3				
Gynoxys hallii Hieron.		1									3				
Monnina phillyreoides (Bonpl.) B. Eriksen		1									3				
Gaultheria amoena A.C. Sm.		1									1	2			
Pentacalia teretifolia (Kunth) Cuatrec.		1													3
Carex sanctae-marthae L.E.Mora & J.O.Rangel			4												
Coespeletia thyrsiformis (A.C.Sm.) Cuatrec.			4												
Diplostephium anactinotum Wedd.			4												
Obtegomeria caerulescens (Benth.) Doroszenko & P.D.Cantino			4												
Oligandra chrysocoma Wedd.			4												
Perissocoeleum purdiei Mathias & Constance			4												

Echeveria bicolor (Kunth) Walth.			1						3						
Laestadia pinifolia Kunth ex Less.				3		1									
Diplostephium spinulosum Wedd.					4										
Hypericum ruscoides Cuatrec.					4										
Ilex laureola Triana					4										
Bromus catharticus Vahl					3				1						
Gynoxys baccharoides (Kunth) Cass.					2						2				
Huperzia rufescens (Hook.) Trevis.					1	1						1	1		
Puya clava-herculis Mez & Sodiro					1			3							
Aragoa occidentalis Pennell						4									
Gnaphalium dysodes Spreng.							3				1				
Hypericum loxense Benth.								3			1				
Sisyrinchium vaginatum Spreng.								2	1		1				
Gynoxys miniphylla Cuatrec.								2			2				
Phlegmariurus hypogaeus (B. Øllg.) B. Øllg.								2			2				
Achyrocline ramosissima Britton									4						
Agrostis mertensii Trin.									4						
Aragoa lucidula S.F.Blake									4						
Bulbostylis capillaris (L.) Kunth ex C.B.Clarke									4						
Elaphoglossum deorsum (H. Karst.) Vareschi									4						
Lachemilla equisetiformis (Trev.) Rothm.									4						
Pityrogramma chrysoconia (Desv.) Maxon ex Domin									4						
Stachys venezuelana Briq.									4						
Valeriana phylicoides (Turcz.) Briq.									4						
Vulpia bromoides (L.) Gray									4						
Draba sericea Santana & Rangel										1			3		
Senecio niveoaureus Cuatrec.										1			3		
Calamagrostis rigida (Kunth) Trin. ex Steud.											4				
Calceolaria hyssopifolia Kunth											4				
Gentianella rupicola (Kunth) Holub											4				
Silene andicola Gillies ex Hook. & Arn.											4				
Aphanactis ollgaardii H. Rob.											2	2			
Ribes hirtum Willd. ex Roem. & Schult.											1	3			
Ranunculus gusmannii Humb. ex Caldas												4			
Senecio hypsobates Wedd.												3		1	
Lasiocephalus gargantanus (Cuatrec.) Cuatrec.												1		3	
Stipa hans-meyeri Pilg.															4
Ruilopezia jahnii (Standl.) Cuatrec.	2			1											
Pentacalia greenmaniana (Hieron.) Cuatrec.	2								1						
Alnus acuminata Kunth		3													
Asplenium serra Langsd. & Fisch.		3													
Begonia urticae L.f.		3													
Calceolaria fusca Pennell		3													
Cuphea ciliata Ruiz & Pav.		3													
Epidendrum erosum Ames & C.Schweinf.		3													
Epidendrum loxense F. Lehm. & Kraenzl.		3													
Eriosorus aureonitens (Hook.) Copel.		3													
Eriosorus cheilanthoides (Sw.) A.F. Tryon		3													
Gynoxys cuicochensis Cuatrec.		3													
Maxillaria klugii C. Schweinf.		3													
Monochaetum discolor H.Karst. ex Triana		3													
Morella parvifolia (Benth.) Parra-O.		3													
Oreopanax bogotensis Cuatrec.		3													
Peperomia tequendamana Trel.		3													
Pitcairnia trianae André		3													
Puya lineata Mez		3													
Sabazia trianae (Hieron.) Longpre		3													
Stelis pusilla Kunth		3													
Stevia andina B.L. Rob.		3													
Tillandsia compacta Griseb.		3													
Tillandsia seemannii (Baker) Mez		3													
Tillandsia tripinnata (Baker) Mez		3													
Tillandsia turneri Baker		3													
Weinmannia cymbifolia Diels		3													
Arcytophyllum capitatum (Benth.) K. Schum.		2			1										
Lycopodiella alopecuroides (L.) Cranfill		2				1									
Eriosorus rufescens (Fée) A.F. Tryon		2							1						
Acaena ovalifolia Ruiz & Pav.		2									1				
Ageratina glechonophylla (Less.) R.M.King & H.Rob.		2									1				
Berberis lobbiana (C.K. Schneid.) C.K. Schneid.		2									1				
Melpomene peruviana (Desv.) A.R. Sm. & R.C. Moran		1	2												

Sisyrinchium chilense Hook.	1	2												
Dryopteris wallichiana (Spreng.) Hyl.	1			1									1	
Juncus effusus L.	1				2									
Blechnum lechleri Mett.	1						2							
Lycopodiella cernua (L.) Pic. Serm.	1						2							
Puya eryngioides André	1						2							
Margyricarpus pinnatus (Lamarck) Kuntze	1						1			1				
Elaphoglossum lindenii (Bory ex Fée) T. Moore	1							2						
Peperomia microphylla Kunth	1							2						
Nassella mucronata (Kunth) R.W.Pohl	1							1						1
Brachyotum jamesonii Triana	1									2				
Chevreulia acuminata Less.	1									2				
Halenia brevicornis (Kunth) G. Don	1									2				
Lamourouxia virgata Kunth	1									2				
Salvia corrugata Vahl	1									2				
Castilleja steyermarkii Pennell		3												
Chaptalia paramensis Cuatrec.		3												
Espeletia boyacensis Cuatrec.		3												
Halenia alata (M. Martens & Galeotti) Hemsl.		3												
Hypericum gleasonii N. Robson		3												
Perissocoeleum phylloideum (Mathias & Constance) Mathias & Constance		3												
Hypericum selaginella N. Robson		2			1									
Geranium subnudicaule Turcz.		1						2						
Miconia parvifolia Cogn.			3											
Senecio formosoides Cuatrec.			3											
Geranium lainzii Aedo			2		1									
Cerastium fontanum Baumg.			1							2				
Baccharis padifolia Hieron.				3										
Gaultheria bracteata (Cav.) G. Don				3										
Greigia exserta L. B. Sm.				3										
Oreopanax ruizanus Cuatrec.				3										
Oxalis phaeotricha Diels				3										
Pentacalia flosfragrans (Cuatrec.) Cuatrec.				1	2									
Jamesonia alstonii A.F. Tryon				1			2							
Moritzia lindenii (A.DC.) Benth. ex Gürke				1				2						
Juncus microcephalus Kunth					3									
Baccharis revoluta Kunth					2				1					
Eleocharis albibracteata Nees & Meyen ex Kunth						3								
Gynoxys fuliginosa (Kunth) Cass.							3							
Oritrophium crocifolium (Kunth) Cuatrec.							3							
Puya pygmaea L.B. Sm.							3							
Valeriana plantaginea Kunth							3							
Aa maderoi Schltr.							2				1			
Berberis discolor Turcz.								3						
Blakiella bartsiifolia (S.F.Blake) Cuatrec.								3						
Cheilanthes marginata Kunth								3						
Conyza mima S.F.Blake								3						
Draba bellardii S.F.Blake								3						
Espeletiopsis pannosa (Standl.) Cuatrec.								3						
Gynoxys moritziana Sch.Bip.ex Wedd.								3						
Monnina meridensis Planch. & Linden ex Wedd.								3						
Nassella depauperata (Pilg.) Barkworth								3						
Pentacalia pachypus (Greenm.) Cuatrec.								3						
Pityrogramma ebenea (L.) Proctor								3						
Polypodium murorum Hook.								2		1				
Vulpia myuros (L) C.C. Gmel.								2		1				
Draba pachythyrsa Triana & Planch.									1			2		
Barkleyanthus salicifolius (Kunth) H.Rob. & Brettell										3				
Calceolaria ericoides Juss. ex Vahl										3				
Geranium antisanae R. Knuth										3				
Gnaphalium chimborazense Rieron.										3				
Festuca parciflora Swallen										2	1			
Poa paramoensis Lægaard										2			1	
Aira caryophyllea L.										2				1
Aetheolaena involucrata (Kunth) B. Nord.										1	2			
Plantago tubulosa Decne.										1	2			
Gentianella sulphurea (Gilg) Fabris											3			
Luzula vulcanica Liebm.											3			
Ranunculus krapfia DC. ex Deless.											3			
Floscaldasia azorelloides Sklenář & H.Rob.													3	
Pentacalia chimborazensis Sklenář														3

Festuca guaramacalana Stancík	2				
Huperzia amentacea (B. Øllg.) Holub	2				
Hymenophyllum trichomanoides Bosch	2				
Ruilopezia viridis (Aristeg.) Cuatrec.	2				
Baccharis phylicoides Kunth		2			
Bartsia sericea Molau		2			
Bartsia tomentosa Molau		2			
Berberis podophylla C. Schneider		2			
Blechnum divergens (Kunze) Mett.		2			
Bomarea dissitifolia Baker		2			
Brachyotum microdon (Naudin) Triana		2			
Brachyotum rostratum (Naudin) Triana		2			
Cacosmia rugosa Kunth		2			
Calceolaria mexicana Benth.		2			
Calceolaria rhododendroides Kraenzl.		2			
Calceolaria tripartita Ruiz & Pav.		2			
Carex larensis Steyerm.		2			
Cavendishia bracteata (Ruiz & Pav. ex J.St.-Hil.) Hoerold		2			
Ceratostema alatum (Hoerold) Sleumer		2			
Chusquea asymmetrica (L.G.Clark) L.G.Clark		2			
Chusquea leonardiorum L.G. Clark		2			
Chusquea loxensis L.G. Clark		2			
Clusia ducuoides Engl.		2			
Culcita coniifolia (Hook.) Maxon		2			
Disterigma codonanthum S.F. Blake		2			
Epidendrum cylindraceum Lindl.		2			
Epidendrum fimbriatum Kunth		2			
Eriocaulon microcephalum Kunth		2			
Eriosorus elongatus (Grev. & Hook.) Copel.		2			
Freziera canescens Humb. & Bonpl.		2			
Fuchsia venusta Kunth		2			
Gaultheria foliolosa Benth.		2			
Geissanthus vanderwerffii Pipoly		2			
Gentianella liniflora (Kunth) Fabris cx J. Pringle		2			
Gynoxys laurifolia (Kunth) Cass.		2			
Hedyosmum racemosum (Ruiz & Pav.) G.Don		2			
Hedyosmum scabrum (Ruiz & Pav.) Solms		2			
Hymenophyllum amabile C. V. Morton		2			
Hymenophyllum dependens C.V. Morton		2			
Ilex myricoides Kunth		2			
Ilex ovalis (Ruiz & Pav.) Loes.		2			
Libanothamnus parvulus Cuatrec.		2			
Lysimachia andina Sandwith		2			
Macrocarpaea bracteata Ewan		2			
Melpomene sodiroi (H. Christ & Rosenst.) A.R. Sm. & R.C. Moran		2			
Miconia dodsonii Wurdack		2			
Miconia poortmannii (Cogn.) Wurdack		2			
Munnozia senecionidis Benth.		2			
Myrsine andina (Mez) Pipoly		2			
Myrsine coriacea (Sw.) R.Br. ex Roem. & Schult.		2			
Myrsine manglilla R.Br.		2			
Niphidium crassifolium (L.) Lellinger		2			
Ocotea sericea Kunth		2			
Paepalanthus andicola Körn.		2			
Psychotria eciliata Steyerm.		2			
Roupala monosperma (Ruiz & Pav.) I.M. Johnst.		2			
Rubus coriaceus Poir.		2			
Rubus gachetensis Berger		2			
Schefflera acuminata (Pav.) Harms		2			
Senecio usgorensis Cuatrecasas		2			
Sticherus lechleri (Mett. ex Kuhn) Nakai		2			
Terpsichore alsopteris (C.V. Morton) A.R. Sm.		2			
Tibouchina laxa (Desr.) Cogn.		2			
Tillandsia wurdackii L.B. Sm.		2			
Utricularia unifolia Ruiz & Pav.		2			
Vriesea tequendamae (André) L.B.Sm.		2			
Weinmannia reticulata Ruiz & Pav.		2			
Ruilopezia hanburiana (Cuatrec.) Cuatrec.		1	1		
Carex acutata Boott		1		1	
Diplostephium empetrifolium S.F. Blake		1			1
Gynoxys lehmannii Hieron.		1			1

Polypodium monosorum Desv.	1			1									
Juncus bufonius L.	1				1								
Miconia tinifolia Naudin	1					1							
Calamagrostis rupestris Trin.	1						1						
Gaultheria lanigera Hook.	1						1						
Gaultheria tomentosa Kunth	1						1						
Grammitis paramicola L. E. Bishop	1						1						
Juncus ecuadoriensis Balslev	1						1						
Valeriana convallarioides (Schmale) B.B. Larsen	1						1						
Campyloneurum amphostenon (Kunze ex Klotzsch) Fée	1							1					
Coriaria ruscifolia L.	1							1					
Dendrophthora meridana Kuijt	1							1					
Diplostephium venezuelense Cuatrec.	1							1					
Drymaria villosa Schltdl. & Cham.	1							1					
Elaphoglossum nivosum (Kunze) Mickel	1							1					
Elaphoglossum tachirense Mickel	1							1					
Myrcianthes myrsinoides (Kunth) F.T.Grifo	1							1					
Poa mucuchachensis Luces	1							1					
Rubus bogotensis Kunth	1							1					
Hydrocotyle gunnerifolia Wedd.	1								1				
Saracha quitensis (Hook.) Miers	1								1				
Aa paleacea (Kunth) Rchb.f.	1									1			
Achyrocline hallii Hieron.	1									1			
Achyrocline trianae Klatt	1									1			
Castilleja virgata (Wedd.) Edwin	1									1			
Galium aparine L.	1									1			
Gynoxys sodiroi Hieron.	1									1			
Phlegmariurus saururus (Lam.) B. Øllg.	1												1
Berberis glauca Kunth		2											
Conyza prolialba Cuatrec.		2											
Lourteigia microphylla (L. f) R. M. King & H. Rob.		2											
Puya grantii L.B.Sm.		2											
Xyris columbiana Malme		2											
Asplenium castaneum Schltdl. & Cham.		1						1					
Oritrophium nevadense (Wedd.) Cuatrec.		1						1					
Uncinia hamata (Sw.) Urb.		1									1		
Gynoxys ilicifolia (L.f.) Wedd.			2										
Paepalanthus lodiculoides Moldenke			2										
Phytolacca icosandra L.			2										
Rhynchospora oreoboloidea Gómez-Laur.			2										
Sisyrinchium convolutum Nocca			2										
Carex livida (Wahlenb.) Willd.			1	1									
Greigia stenolepis L. B. Sm.			1		1								
Aetheolaena otophora (Wedd.) B. Nord.				2									
Aetheolaena patens (Kunth) B. Nord.				2									
Geranium rhomboidale H.E. Moore				2									
Jamesonia scammaniae A.F. Tryon				2									
Tibouchina grossa (L.f.) Cogn.				2									
Plagiocheilus solivaeformis DC.				1					1				
Calamagrostis densiflora (J. Presl) Steud.					2								
Hypericum prostratum Cuatrec.					2								
Juncus cyperoides Laharpe					1	1							
Cerastium kunthii Briq.					1					1			
Gentianella limoselloides (Kunth) Fabris						1				1			
Diplostephium oblanceolatum S.F. Blake							2						
Gentianella saxifragoides (Kunth) Fabris							2						
Hieracium frigidulans Zahn							2						
Huperzia capellae (Herter) Holub							2						
Huperzia compacta (Hook.) Trevis.							2						
Lupinus revolutus C.P. Sm.							2						
Miconia prietoi Wurdack							2						
Sporobolus lasiophyllus Pilg.							2						
Uncinia paludosa G.A. Wheeler & Goetgh.							1			1			
Vicia setifolia Kunth							1			1			
Senecio tephrosioides Turcz.							1				1		
Viola glandularis H.E. Ballard & P. Jorg.							1				1		
Asplenium castaneum Schltdl. & Cham.								2					
Asplenium polyphyllum Bertol.								2					
Calamagrostis meridensis (Luces) Briceño								2					
Coespeletia spicata (Sch.Bip. ex Wedd.) Cuatrec.								2					
Draba arbuscula Hook.f.								2					

Drymaria ovata Willd. ex Roem. & Schult.		2						
Erigeron paramensis Aristeg. & Cuatrec.		2						
Gynoxys meridana Cuatrec.		2						
Hieracium venezuelanum Arvet-Touvet		2						
Lupinus peruvianus Ulbr.		2						
Lupinus pygmaeus Tamayo ex Pittier		2						
Pentacalia imbricatifolia (Sch.Bip. ex Wedd.) Cuatrec.		2						
Ruilopezia floccosa (Standl.) Cuatrec.		2						
Senecio aristeguietae Cuatrec.		2						
Senecio funckii Sch.Bip.		2						
Setaria parviflora (Poir.) Kerguélen		2						
Stevia caracasana DC.		2						
Urtica ballotifolia Wedd.		1	1					
Gnaphalium domingense Lam.			1			1		
Hymenophyllum karstenianum J. W. Sturm			1			1		
Ageratina sodiroi (Hieron.) R.M. King & H. Rob.				2				
Aristeguietia glutinosa (Lam.) R.M. King & H. Rob.				2				
Asplenium peruvianum Desv.				2				
Bidens rubifolia Kunth				2				
Geranium sericeum Willd. ex Spreng.				2				
Nassella brachyphylla (Hitchc.) Barkworth				2				
Oxalis filiformis Kunth				2				
Sagina apetala Ard.				2				
Sonchus oleraceus L.				2				
Calamagrostis teretifolia Lægaard				1	1			
Cuatrecasasiella isernii (Cuatrec.) H. Rob.				1	1			
Draba pycnophylla Turcz.				1				1
Cerastium crassipes Bartl.					2			
Draba alyssoides Humb. & Bonpl. ex DC.					2			
Draba hammenii Cuatrec. & Cleef						1	1	
Draba extensa Wedd.							2	
Hypolepis obtusata (C. Presl) Hieron.							2	
Phlegmariurus tetragonus (Hook. & Grev.) B. Øllg.							2	
Acalypha diversifolia Jacq.	1							
Acalypha macrostachya Jacq.	1							
Acalypha padifolia Kunth	1							
Ageratina piurae (B. Rob.) King & H. Rob.	1							
Ageratina pseudochilca (Benth.) R.M. King & H. Rob.	1							
Ageratina scopulorum (Wedd.) R. M. King & H. Rob.	1							
Agrostis subrepens (Hitchc.) Hitchc.	1							
Alansmia dependens (Baker) Moguel & M. Kessler	1							
Antennaria linearifolia Wedd.	1							
Anthurium oxybelium Schott	1							
Antidaphne andina Kuijt	1							
Axinaea macrophylla (Naudin) Triana	1							
Axinaea nitida Cogn.	1							
Axonopus fissifolius (Raddi) Kuhlm.	1							
Baccharis bogotensis Kunth	1							
Bartsia trichophylla Wedd.	1							
Bartsia weberbaueri Diels	1							
Berberis lutea Ruiz & Pav.	1							
Bomarea angulata Benth.	1							
Bomarea nervosa (Herb.) Baker	1							
Brachyotum fraternum Wurdack	1							
Brachyotum grisebachii Cogn.	1							
Brachyotum tyrianthinum J. F. Macbr.	1							
Bulbostylis juncoides (Vahl) Kök. ex Osten	1							
Cardamine alberti O. E. Schulz	1							
Castilleja meridensis Pennell	1							
Cavendishia bracteata (Ruiz & Pav. ex J.St.-Hil.) Hoerold	1							
Ceradenia meridensis (Klotzsch) L.E. Bishop	1							
Ceratostema reginaldii (Sleumer) A.C. Sm.	1							
Chusquea neurophylla L.G. Clark	1							
Chusquea perligulata (Pilg.) McClure	1							
Chusquea scandens Kunth	1							
Cinchona mutisii Lamb.	1							
Clethra cuneata Rusby	1							
Clethra fagifolia Kunth	1							
Clinopodium revolutum (Ruiz & Pav.) Govaerts	1							
Clinopodium taxifolium (Kunth) Govaerts	1							
Clusia multiflora Kunth	1							

Cyathea parvifolia Sodiro	1
Cybianthus pastensis (Mez) G. Agostini	1
Cynanchum tenellum L. f.	1
Dendrophthora lindeniana Tiegh.	1
Diplostephium foliosissimum S. F. Blake	1
Diplostephium jelskii Hieron.	1
Ditassa longiloba Benth.	1
Elaeagia ecuadorensis Steyerm.	1
Elaphoglossum antisanae (Sodiro) C. Chr.	1
Elaphoglossum dendricola (Baker) H. Christ	1
Elaphoglossum melancholicum Vareschi	1
Elaphoglossum tectum (Humb. & Bonpl. ex Willd.) T. Moore	1
Epidendrum frigidum Linden ex Lindl.	1
Epidendrum macrostachyum Lindl.	1
Escallonia paniculata (Ruiz & Pav.) Roem. & Schult.	1
Euphorbia repens K. Koch	1
Fernandezia crystallina (Lindl.) M.W.Chase	1
Fernandezia theodorii M.W. Chase	1
Frangula granulosa (Ruiz & Pav.) Grubov.	1
Freziera karsteniana (Szyszył.) Kobuski	1
Gaultheria vaccinioides Griseb. ex Wedd.	1
Gentianella oellgaardii J.S. Pringle	1
Gentianella setipes (Gilg) J. Pringle	1
Geonoma orbignyana Mart.	1
Geranium chilloense Willd. ex Kunth	1
Geum peruvianum Focke	1
Graffenrieda harlingii Wurdack	1
Gunnera pilosa Kunth	1
Guzmania squarrosa (Mez & Sodiro) L.B.Sm. ex Pittendr.	1
Halenia gigantea C.K.Allen	1
Halenia sphagnicola Gilg	1
Hedyosmum cumbalense H. Karst.	1
Hedyosmum goudotianum Solms	1
Hedyosmum sprucei Solms	1
Heppiella verticillata (Cav.) Cuatrec.	1
Hieronyma duquei Cuatrec.	1
Huperzia binervia (Herter) B. Øllg.	1
Huperzia reflexa (Lam.) Trevis	1
Hydrocotyle hitchcockii Rose ex Mathias	1
Hyeronima macrocarpa Müll. Arg.	1
Hypericum chamaemyrtus Triana & Planch.	1
Hypochaeris chillensis (Kunth) Hieron.	1
Ilex andicola Loes.	1
Ilex gabinetensis Cuatrec.	1
Ilex rimbachii Standl.	1
Juncus arcticus Willd.	1
Lellingeria major (Copel.) A.R. Sm. & R.C. Moran	1
Lepanthes flexuosa Luer	1
Lycopodium vestitum Desv. ex Poir.	1
Malaxis andicola (Ridl.) Kuntze	1
Meriania furvanthera Wurdack	1
Meriania maguirei Wurdack	1
Meriania rigida (Benth.) Triana	1
Mezobromelia fulgens L.B. Sm.	1
Miconia hutchisonii Wurdack	1
Miconia squamulosa Triana	1
Miconia theaezans (Bonpl.) Cogn.	1
Mikania stuebelii Hieron.	1
Monnina conferta Ruiz & Pav.	1
Monnina decurrens Ferreyra	1
Monnina hirta (Bonpl.) B. Eriksen	1
Monticalia pulchella (Kunth) C. Jeffrey	1
Myrcianthes rhopaloides (Kunth) McVaugh	1
Myrteola acerosa (O. Berg) Burret	1
Nassella pubiflora (Trin. & Rupr.) E. Desv.	1
Ocotea infrafoveolata van der Werff	1
Oreocallis grandiflora (Lam.) R. Br.	1
Oreopanax discolor (Kunth) Decne. & Planch.	1
Oreopanax rosei Harms	1
Oritrophium repens (Kunth) Cuatrec.	1
Oxalis fendleri Lourteig	1

Panicum pantrichum Hack.	1			
Paspalum candidum (Flüggé) Kunth	1			
Passiflora cumbalensis (H. Karst.) Harms	1			
Pennisetum bambusiforme (E.Fourn.) B.D.Jacks.	1			
Pennisetum clandestinum Hochst. ex Chiov.	1			
Peperomia rotundata Kunth	1			
Persea brevipes Meisn.	1			
Persea bullata Kopp	1			
Pityrogramma pearcei (T. Moore) Domin	1			
Plagiogyria semicordata (C. Presl) H. Christ	1			
Podocarpus oleifolius D. Don	1			
Polygala paniculata L.	1			
Polylepis weberbaueri Pilg.	1			
Prunus opaca (Benth.) Walp.	1			
Puya bicolor Mez	1			
Puya exigua Mez	1			
Puya glaucovirens Mez	1			
Rhynchospora glomerata (L.) Vahl	1			
Rhynchospora polyphylla (Vahl) Vahl	1			
Rhynchospora talamancensis Gómez-Laur. & W. W. Thomas	1			
Ribes bogotanum Jancz.	1			
Ribes ecuadorense Jancz.	1			
Ribes microphyllum Kunth	1			
Rubus floribundus Kunth	1			
Rubus laegaardii Romoleroux	1			
Rubus megalococcus Focke	1			
Rubus robustus C. Presl	1			
Smilax domingensis Willd. Kunth	1			
Sphyrospermum cordifolium Benth.	1			
Stelis concinna Lindl.	1			
Stenomesson aurantiacum (Kunth) Herb.	1			
Stevia elatior Kunth	1			
Sticherus simplex (Desv.) Ching	1			
Themistoclesia epiphytica A.C. Sm.	1			
Tillandsia biflora Ruiz & Pav.	1			
Tillandsia complanata Benth.	1			
Trachypogon spicatus (L.f.) Kuntze	1			
Triglochin scilloides (Poir.) Mering & Kadereit	1			
Valeriana hirtella Kunth	1			
Vitekorchis excavata (Lindl.) Romowicz & Szlach.	1			
Weinmannia anisophylla Standl. & L.O. Williams	1			
Weinmannia jelskii Zahlbr.	1			
Weinmannia karsteniana Szyszyl.	1			
Weinmannia lechleriana Engl.	1			
Weinmannia pubescens Kunth	1			
Woodsia montevidensis (Spreng.) Hieron.	1			
Aa leucantha (Rchb.f.) Schltr.		1		
Diplostephium rangelii Cuatrec.		1		
Diplostephium weddellii S. F. Blake		1		
Halenia elata Wedd.		1		
Monnina aestuans (L.f.) DC.		1		
Niphogeton colombiana Mathias & Constance		1		
Pentacalia carrikeri (Cuatrec.) Cuatrec.		1		
Pentacalia weinmannifolia (Cuatrec.) Cuatrec.		1		
Senecio subruncinatus (Wedd.) Greenm.		1		
Symplocos rigidissima Brand		1		
Valeriana engleriana Höck		1		
Valeriana karstenii Briq.		1		
Valeriana vetasana Killip		1		
Aragoa cupressina Kunth			1	
Aulonemia trianae (Munro) McClure			1	
Chusquea acuminatissima (Munro) L.G.Clark (Munro) Pilg.			1	
Elaphoglossum affine (M. Martens & Galeotti) T. Moore			1	
Epidendrum chortophyllum Schltr.			1	
Fleischmannia pycnocephala (Less.) R.M.King & H.Rob.			1	
Frangula goudotiana (Triana & Planch.) Grubov			1	
Lupinus bogotensis Benth.			1	
Miconia nivalis Wurdack			1	
Scrobicaria ilicifolia (L. f.) B. Nord.			1	
Alansmia lanigera (Desv.) Moguel & M. Kessler				1
Berberis stuebelii Hieron.				1

Cardamine ovata Benth.	1				
Carex stricta Lam.	1				
Disterigma humboldtii (Klotzsch) Nied.	1				
Drymaria cordata (L.) Willd. ex Roem. & Schult.	1				
Elaphoglossum cuspidatum (Willd.) T. Moore	1				
Ilex colombiana Cuatrec.	1				
Jamesonia robusta H. Karst.	1				
Myosotis azorica H. C. Watson	1				
Oreopanax ellsworthii Cuatrec.	1				
Pentacalia leioclada (Cuatrec.) Cuatrec.	1				
Ribes leptostachyum Benth.	1				
Schefflera ferruginea (Willd. ex Schult.) Harms	1				
Symplocos venulosa Cuatrec.	1				
Valeriana tatamana Killip	1				
Berberis psilopoda Turcz.		1			
Espeletia barclayana Cuatrec.		1			
Espeletia incana Cuatrec.		1			
Espeletia summapacis Cuatrec.		1			
Espeletia uribei Cuatrec.		1			
Huperzia dianae (Herter) B. Øllg.		1			
Lachemilla pinnata (Ruiz & Pav.) Rothm.		1			
Niphogeton cleefii Mathias & Constance		1			
Niphogeton josei Mathias & Constance		1			
Pentacalia ledifolia (Kunth) Cuatrec.		1			
Ribes columbianum Cuatrec.		1			
Valeriana stenophylla Killip		1			
Baccharis teindalensis Kunth			1		
Uncinia tenuis Poepp. ex Kunth			1		
Belonanthus hispida (Wedd.) Graebn.				1	
Chusquea nana (L.G.Clark) L.G.Clark				1	
Halenia umbellata (Ruiz & Pav.) Gilg				1	
Huperzia columnaris B. Øllg.				1	
Isidrogalvia sessiliflora (Hook.) Cruden				1	
Lysipomia vitreola McVaugh				1	
Miconia rotundifolia (D. Don) Naudin				1	
Oxalis eriolepis Wedd.				1	
Acaulimalva acaulis (Dombey ex Cav.) Krapov.					1
Ageratina aristeguietii R. M. King & H. Rob.					1
Agrostis meridensis Luces					1
Agrostis venezuelana Mez					1
Alloispermum caracasanum (Kunth) H. Rob.					1
Altensteinia nubigena (Rchb.f.) Rchb.f.					1
Athyrium filix-femina (L.) Roth					1
Berberis prolifica Pittier					1
Blakiella bartsiifolia (S.F.Blake) Cuatrec.					1
Calamagrostis chaseae Luces					1
Calceolaria perfoliata L.f.					1
Carex phalaroides Kunth					1
Castilleja trujillensis Pennell					1
Draba chionophila S. F. Blake					1
Draba funckiana Planch. ex O.E.Schulz					1
Elaphoglossum appressum Mickel					1
Elaphoglossum dombeyanum (Fée) T.B. Moore & Houlston					1
Espeletia aurantia Aristeg.					1
Espeletia semiglobulata Cuatrec.					1
Gamochaeta paramora (S.F.Blake) Anderb.					1
Gomphichis traceyae Rolfe					1
Gomphichis viscosa (Rchb.f.) Schltr.					1
Halenia subinvolucrata Gilg					1
Hieracium erianthum Kunth					1
Hierochloe redolens (Vahl) Roem. & Schult.					1
Hymenophyllum polyanthos Sw.					1
Hypericum thesiifolium Kunth					1
Lappula squarrosa (Retz.) Dumort.					1
Lupinus jahnii Rose ex Pittier					1
Lysipomia bourgoinii Ernst					1
Oreopanax reticulatus (Willd ex Schult) Decne & Planch. ME					1
Oritrophium venezuelense (Steyerm.) Cuatrec.					1
Pentacalia sclerosa (Cuatrec.) Cuatrec.					1
Pentacalia tunamensis (Cuatrec.) Cuatrec.					1
Phoradendron undulatum (Pohl ex DC.) Eichler					1

Polylepis sericea Wedd.	1					
Ranunculus bonariensis Poir.	1					
Ribes canescens Pittier	1					
Salvia rubescens Kunth	1					
Serpocaulon lasiopus (Klotzsch) A.R. Sm.	1					
Siphocampylus sceptrum Decne. ex Linden	1					
Solanum colombianum Dunal	1					
Thalictrum podocarpum Kunth ex DC.	1					
Vaccinium meridionale Sw.	1					
Valeriana scandens Loefl.	1					
Calceolaria penlandii Pennell		1				
Cardamine jamesonii Hook.		1				
Aetheolaena pichinchensis (Cuatrec.) B. Nord.			1			
Asplenium oellgaardii Stolze			1			
Bartsia pumila Benth.			1			
Berberis multiflora Benth.			1			
Bomarea perglabra Harling & Neuendorf			1			
Bowlesia lobata Ruiz & Pav.			1			
Caiophora contorta (Desr.) C. Presl			1			
Calceolaria crenata Lam.			1			
Calceolaria lamiifolia Kunth			1			
Carex umbellata Willd.			1			
Descurainia myriophylla (Willd. ex DC.) R.E. Fr.			1			
Diplostephium glutinosum S.F.Blake			1			
Elaphoglossum paleaceum (Hook. & Grey.) Sledge			1			
Ephedra americana Humb. & Bonpl. ex Willd.			1			
Festuca densipaniculata E.B. Alexeev			1			
Festuca sodiroana Hack. ex E.B. Alexeev			1			
Gnaphalium cheiranthifolium Bertero ex Lam.			1			
Gnaphalium coarctatum Willd.			1			
Hieracium sprucei Arv.-Touv.			1			
Huperzia lindenii (Spring) Trevis.			1			
Hypericum caespitosum Cham. & Schltdl.			1			
Jungia rugosa Less.			1			
Lachemilla paludicola (Rothm.) Rothm.			1			
Lupinus ulbrichianus C.P. Sm.			1			
Lysipomia acaulis Kunth			1			
Muhlenbergia peruviana (P. Beauv.) Steud.			1			
Oxalis corniculata L.			1			
Oxalis mollis Kunth			1			
Plantago lanceolata L.			1			
Poa aequatoriensis Hack.			1			
Polylepis reticulata Hieron.			1			
Potentilla dombeyi Nestl.			1			
Taraxacum vulgare (Lam.) Schrank			1			
Urtica leptophylla Kunth			1			
Veronica arvensis L.			1			
Vulpia australis (Nees) Blom			1			
Aetheolaena caldasensis (Cuatrec.) B.Nord.				1		
Aphanactis barclayae H. Rob.				1		
Chusquea villosa (L.G.Clark) L.G.Clark				1		
Draba spruceana Wedd.				1		
Festuca imbaburensis Stancík				1		
Huperzia hohenackeri (Herter) Holub				1		
Isoetes killipii C. V. Morton				1		
Lachemilla perryana (Rothm.) Rothm.				1		
Lasiocephalus puracensis (Cuatrec.) Cuatrec.				1		
Myrosmodes nubigenum Rchb.f.				1		
Oritrophium llanganatense Sklenár & H. Rob.				1		
Rostkovia magellanica (Lam.) Hook. f.				1		
Poa trivialis L.					1	
Arenaria tetragyna Willd. ex Schltdl.						1
Elaphoglossum rimbachii (Sodiro) H. Christ						1
Senecio rufescens DC.						1
